# Filamented hydrogels as tunable conduits for guiding neurite outgrowth

**DOI:** 10.1016/j.mtbio.2025.101471

**Published:** 2025-01-11

**Authors:** Hao Liu, Anna Puiggalí-Jou, Parth Chansoria, Jakub Janiak, Marcy Zenobi-Wong

**Affiliations:** Tissue Engineering + Biofabrication Laboratory, Department of Health Sciences & Technology, ETH Zurich, Otto-Stern-Weg 7, Zürich, 8093, Switzerland

**Keywords:** Filamented light, Dorsal root ganglion, Neurite alignment, Nerve growth factor

## Abstract

Anisotropic scaffolds with unidirectionally aligned fibers present an optimal solution for nerve tissue engineering and graft repair. This study investigates the application of filamented light (FLight) biofabrication to create hydrogel matrices featuring highly aligned microfilaments, facilitating neurite guidance and outgrowth from encapsulated chicken dorsal root ganglion (DRG) cells. FLight employs optical modulation instability (OMI) to rapidly and safely (<5 s) fabricate hydrogel constructs with precise microfilament alignment. The tunability of FLight matrices was demonstrated by adjusting four key parameters: stiffness, porosity, growth factor release, and incorporation of biological cues. Matrix stiffness was fine-tuned by varying the projection light dose, yielding matrices with stiffness ranging from 0.6 to 5.7 kPa. Optimal neurite outgrowth occurred at a stiffness of 0.6 kPa, achieving an outgrowth of 2.5 mm over 4 days. Matrix porosity was modified using diffraction gratings in the optical setup. While significant differences in neurite outgrowth and alignment were observed between bulk and FLight gels, further increases in porosity from 40 % to 70 % enhanced cell migration and axon bundling without significantly affecting maximal outgrowth. The incorporation of protein microcrystals containing nerve growth factor (NGF) into the photoresin enabled sustained neurite outgrowth without the need for additional NGF in the media. Finally, laminin was added to the resin to enhance the bioactivity of the biomaterial, resulting in a further increase in maximum neurite outgrowth to 3.5 mm after 4 days of culture in softer matrices. Overall, the varied matrix properties achieved through FLight significantly enhance neurite outgrowth, highlighting the importance of adaptable scaffold characteristics for guiding neurite development. This demonstrates the potential of FLight as a versatile platform for creating ideal matrices for clinical applications in nerve repair and tissue engineering.

## Highlights

• Rapid production (within seconds) of biologically relevant anisotropic constructs for neural tissue regeneration.
• Filamented Light (FLight) biofabricated scaffolds effectively guide neurite outgrowth.
• Versatile platform with tunable stiffness, microarchitecture, matrix composition, and growth factor delivery.
• Neurite extends 3.5 mm during 4 days of *in vitro* culture.

## Graphical abstract

**Figure:**
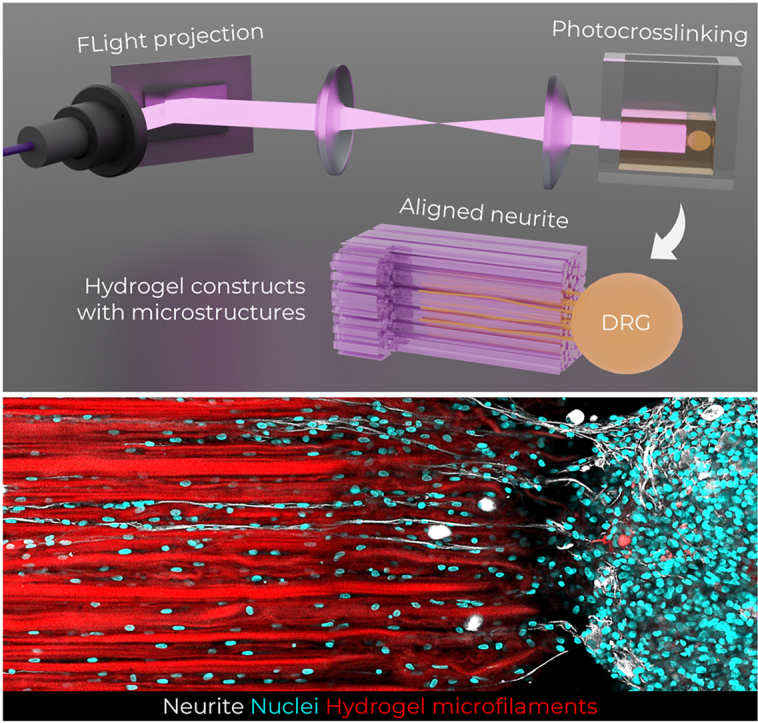

## 1 Introduction

The aligned microarchitecture of the nerves is a fundamental aspect of efficient physiological signal transmission for coordinated functions of both the peripheral and central nervous systems [1]. In peripheral nerves, axons are bundled into myelinated and non-myelinated fibers, forming the endoneurium. These bundles are surrounded by the perineurium, creating fascicles. Multiple fascicles are then enclosed by the epineurium, completing the hierarchical structure of peripheral nerves [2]. A disruption in the neuronal synapses, such as after an injury, can often result in the loss of motor or sensory function. This may lead to long-term or permanent debilitation and socioeconomic burden [3,4]. In cases of mild injuries, nerve function can return spontaneously; however, in critical-sized defects where the nerve is severed, surgical intervention, such as direct anastomosis, biological grafts, or nerve conduits, is needed. Significant clinical challenges include the presence of finger defects exceeding 10 mm and hand and foot defects exceeding 30 mm [5].

Currently, autograft nerves are considered the gold standard for surgical treatment due to their superior functional recovery compared to commercially available products like nerve conduits or acellular nerve allografts. Autografts provide regenerating host axons with anisotropic structural support and are rich in growth factors secreted by Schwann cells. Despite the advantages of autografts, they face challenges such as donor site comorbidities and limited availability [6]. Nerve allografts, as an alternative, present no donor site morbidity for the patient, possess the right mechanical properties (i.e., facilitating handling), and are widely available (e.g., Avance® graft from Axogen, which is a commonly used graft for peripheral nerve repair). However, there are concerns about histocompatibility and ethics. Finally, nerve conduits from engineered materials are easy to produce, there is no donor site trauma, and there are unlimited resources. Examples include collagen type I-based NeuraGen®, NeuroMatrix™ and Neuroflex™ conduits, polyglycolic acid (PGA)-based Neurotube® conduit, or Poly(dl-lactide-ɛ-caprolactone) (PCL)-based Neurolac® conduit. These conduits are up to 10 mm in diameter and up to 6.5 cm in length. Nerve grafts have significantly advanced repair strategies, yet they still struggle to support robust nerve regeneration, especially across large-diameter and distance gaps. Most current grafts are empty conduits lacking essential components such as extracellular matrix, growth factors, and supportive cells, which limits their regenerative potential and slows healing [7]. There is, therefore, an urgent need for a transformational technique that will redefine the way that biomimetic nerve grafts are fabricated.

Recent studies have demonstrated remarkable progress in developing anisotropic scaffolds to promote axonal growth and guidance [1,8]. These scaffolds have been developed from natural or synthetic polymers and processed using various techniques, including freeze-drying [9,10], electrospinning [11], melt electrowriting [12], magnetically assisted fibers/microgels alignment [13,14], and additive manufacturing methods such as micro-extrusion printing or digital light projection [15]. Unfortunately, all these techniques have low throughput (graft fabrication typically takes several minutes to hours) and thus limited scalability. Furthermore, anisotropy alone is insufficient to facilitate rapid and effective repair of large-sized defects. An ideal nerve graft scaffold should facilitate rapid nerve guidance and bridging, as a slow regenerative process cannot effectively counteract Wallerian degeneration [16], potentially leading to suboptimal outcomes in nerve repair. To increase their effectiveness, grafts have also been augmented with cells (e.g., Schwann cells and endothelial cells) [17,18], growth factors (e.g., NGF) [19], and bioactive molecules (e.g., laminin and fibronectin) [20]. These advancements hold promise for creating new clinical grafts that enhance nerve regeneration and functional recovery, gradually reducing the need for autografts (e.g., sural nerves), which are not suitable for larger defects and lead to morbidity (e.g., loss in sensory function) of the donor site.

Filamented Light (FLight) biofabrication generates microfilament-containing hydrogel matrices that provide 3D topological cues for neurite guidance and outgrowth within seconds. FLight printing uses optical modulation instability (OMI) [21,22] and the self-focusing effect within a nonlinear medium (e.g., photocrosslinkable biomaterials) to create hydrogel constructs with highly aligned microfilaments. Specifically, when a customized light pattern is projected onto bioresin where cells are homogenously suspended, the cells are rapidly and safely (>95 % cell viability) encapsulated in 3D hydrogel. Within the hydrogel, the distribution of local light intensity maxima and minima results in crosslinking of microfilaments and formation of channel-like void spaces (microchannels) after washing. Since the size of the cell is larger than those microchannels, the encapsulated cells become entrapped within the hydrogel constructs consisting of microfilaments and microchannels. Our previous work has shown that these cells interact with the microstructures. For example, cells will migrate to neighboring microchannels, accompanied by cell deformation, then highly aligned along the direction of microfilaments. Additionally, the microchannels can effectively support cell proliferation, differentiation, and extracellular matrix (ECM) deposition, thus enabling the biomimicry of anisotropic tissues such as muscle, tendon, and cartilage [23,24].

Here, we demonstrate that the microfilaments and respective microchannels (ɸ ∼2–10 μm) present in FLight hydrogels successfully guide neurite outgrowth and alignment from chicken dorsal root ganglia (DRGs) [25]. This finding potentially opens up new applications for the FLight matrices as scaffolds to regenerate nerve defects and as tissue models of nerve-related injuries. Additionally, we capitalize on the flexibility and versatility of FLight biofabrication by modulating four key parameters: (i) matrix stiffness (0.6–5.7 kPa) by changing the projection light dose, (ii) matrix porosity (40–70 %) by placing diffraction gratings in optical setup, (iii) nerve growth factor availability using protein-microcrystals in the photoresin and (iv) bioactivity of the FLight matrices by adding additional laminin. In each condition, we investigate the process of neurite outgrowth, specifically the length of the neurite, the number of intersections, and their alignment. Finally, we highlight future requirements for the clinical translation of FLight matrices as clinical grafts for peripheral nerve injury repair and discuss the development of models of neural injuries and diseases.

## 2 Materials and methods

### 2.1 Synthesis of gelatin-norbornene (Gel-NB) and thiol-functionalized gelatin (Gel-SH)

The synthesis protocol of Gel-NB and Gel-SH was adapted from previous work [26]. Briefly, for Gel-NB synthesis, 25 g of gelatin type A from porcine skin was dissolved in 0.3 M carbonate-bicarbonate buffer at 40 °C to prepare 10 % w/v gelatin solution. After fully dissolving the gelatin, 0.5 g of cis-5-norbornene-endo-2,3-dicarboxylic anhydride (CA) crystalline solid was added to the reaction mixture under vigorous stirring. CA crystalline solid was sequentially added for a total amount of 2.0 g. The reaction solution was diluted two-fold in pre-warmed milliQ (mQ) H_2_O, and the pH was adjusted to 7 before filtration (0.2 μm pore size). The solution was dialyzed (3.5 kDa cutoff cellulose tubing) for 3–4 days against mQ H_2_O at 37 °C with frequent water changes before lyophilization. The degree of functionalization (DoF) was calculated using ^1^H NMR (Bruker Ultrashield 400 MHz, 1024 scans). The DoF of Gel-NB estimated to be approximately 45 % (≈ 0.091 mmol g^−1^), was calculated as a percentage was calculated based on the lysine and hydroxylysine content of gelatin as previously reported [27].

For Gel-SH synthesis, 25 g of gelatin type A was first dissolved in 1.25 L of 150 mM MES buffer (pH = 4.0) warmed up to 40 °C for a final concentration of 2 % w/v. When completely dissolved, 2.38 g (10 mmol) of 3,3′-dithiobis(proprionohydrazide) (DTPHY) was added to the reaction solution under stirring. Then, 3.83 g (20 mmol) of 1-ethyl-3-(3′-dimethylaminopropyl)carbodiimide hydrochloride (EDC) was added, and the reaction was left to proceed overnight at 40 °C. Tris(2-carboxyethyl)phosphine (8.6 g, 30 mmol) was then added to the reaction mixture, and the reaction was left to proceed for 12 h in a sealed flask under gentle stirring. The solution was filtered (0.2 μm) and dialyzed (3.5 kDa cutoff cellulose tubing) for 3–4 days against mQ H_2_O balanced to pH 4.5 with diluted HCl at 37 °C before lyophilization. Gel-SH was stored under an inert atmosphere at −20 °C prior to use. The DoF of Gel-SH was determined by ^1^H NMR (Bruker Ultrashield 400 MHz, 1024 scans), and found to be ≈ 38 % (0.276 mmol g^−1^).

To synthesize Gelatin-Rhodamine (Gel-Rho), 1.0 g of Gel-NB was dissolved in 100 mL of 0.3 m sodium bicarbonate (pH = 9.0) at 37 °C overnight. 10.0 mg of rhodamine B isothiocyanate was dissolved in 1.0 mL dimethyl sulfoxide (DMSO) and added to the Gel-NB solution. The reaction was left to proceed at room temperature overnight. The product was purified by dialysis (3.5 kDa cut-off) against mQ H_2_O at 40 °C for 3 days with frequent water changes, protected from light, and then lyophilized.

### 2.2 Dorsal root ganglia (DRG) isolation and FLight printing

DRGs were isolated from 8 days-old chicken embryos (E8) [28]. The DRGs were carefully washed with pre-warmed 1 × PBS and transferred to Neurobasal medium (ThermoFisher 21103049) supplemented with 0.5 mM GlutaMAX™ (ThermoFisher 35050038), 2 % of B-27™ (ThermoFisher 17504044), and 20 ng/ml Mouse NGF 7S subunit native protein (ThermoFisher 132900-010).

The lyophilized Gel-NB and Gel-SH were dissolved in 1 × PBS at 37 °C with 1:1 M ratio of NB to SH. Photoinitiator lithium phenyl-2,4,6-trimethylbenzoylphosphinate (LAP) was added from a stock solution of 5 % w/v in 1 × PBS to obtain a final concentration of 0.05 % w/v. Photoresins were prepared with a total polymer concentration of 3 % w/v and filtered through 0.2 μm filters to remove scattering particles and sterilize photoresin prior to use. The additional components such as laminin (Sigma-Aldrich L2020) and PODS® (Cell Guidance Systems PPH316; PPH303) were mixed/resuspended in photoresin at different concentrations according to study designs.

200 μL photoresin was first loaded into cuvettes (4 mm of inner diameter along the projection direction). Freshly extracted DRGs were then transferred to the photoresin and positioned at the end of the projection direction using a sterilized spatula. This positioning ensured that the DRGs were encapsulated on one side of the hydrogel construct, allowing for the neurite outgrowth in a single direction. The cuvettes were transferred to 4 °C for 15 min. The FLight printing process was performed with a customized FLight printer (Lasertack 500 mW 405 nm Diode Laser, and Raspberry Pi with integrated control software), with 55 mW/cm^2^ laser intensity and 200–300 mJ/cm^2^ light dose. The printer was equipped with motorized filter flip mounts (Thorlabs MFF101) and metal meshes as photomask (BOPP 10046 series). The metal mesh (w63) consists of a square grid pattern with each square having a side length of 63 μm. The spacing between each grid square, defined as the distance from the edge of one square to the edge of the adjacent square, is 35 μm. The w90 metal mesh features squares with a side length of 90 μm and the same 35 μm spacing between them. The projection image was designed as a rectangle (2 × 6 mm) using Affinity Photo software with a resolution of 1920 × 1080 pixels in 8-bit grayscale (in PNG format). The projection image was then transferred to the FLight printer and loaded onto the built-in digital micromirror device (DMD) controller for printing. The dimension of the projection area exceeds the diameter of the DRGs (approximately 500 μm), ensuring that the DRGs can be fully encapsulated within 3D FLight hydrogel constructs. Right after the FLight printing, the cuvettes were warmed up in a heating bath at 37 °C. The uncrosslinked photoresin was then removed with 2–3 times washing in pre-warmed 1 × PBS. The DRG-laden hydrogel constructs were transferred to Neurobasal medium for 4 days of culture.

### 2.3 Immunofluorescent staining, confocal imaging and 3D reconstruction

After four days of culturing, the culture medium was removed from the DRG-laden FLight hydrogel samples and washed with pre-warmed 1 × PBS three times. The constructs were then fixed in 4 % paraformaldehyde (PFA) solution for 30 min at room temperature. Subsequently, the hydrogel samples were washed three times with 1 × PBS and treated with 0.2 % v/v Triton X-100 in 1 × PBS for 30 min for permeabilization, prior to being blocked with 1 % w/v bovine serum albumin in 1 × PBS (BSA-PBS) for 2 h. This was followed by incubation with the primary anti-beta III tubulin antibody (abcam 18207, 1:300 dilution) in 1 % BSA-PBS solution 12 h at 4 °C with gentle shaking at 20 rpm. After washing 1 × PBS three times, samples were incubated with 1:500 diluted secondary antibodies, 1:1000 diluted Hoechst 33342, and pre-prepared phalloidin-tetramethylrhodamine B isothiocyanate working solution (0.13 μg mL^−1^, Sigma-Aldrich P1951) in 1 % w/v BSA-PBS for 2 h at 4 °C.

To confirm the encapsulation and presence of laminin (LN, Sigma-Aldrich L2020), fibronectin (FN, Sigma-Aldrich F1056) and their mixture (FN-LN), the laminin stock solution was diluted in fluorescently labeled Gel-NB (Gel-Rho) with a final concentration of 37.5 μg/mL (Low LN) and 75.0 μg/mL (High LN) to prepare photoresin. Similarly, the fibronectin stock solution and FN-LN mixture was added to Gel-Rho with a final concentration of 75 μg/mL. Following the FLight printing, the constructs were initially washed with 1 × PBS to remove uncrosslinked photoresin and fixed in 4 % PFA for 30 min. Next, the printed hydrogel matrices were incubated with primary anti-laminin polyclonal antibody (ThermoFisher PA1-16730, 1:500 dilution) and primary anti-fibronectin antibody (R&D systems MAB1918, 1:200 dilution). The primary antibody solution was then exchanged with fresh 1 × PBS three times and incubated with secondary antibody (Alexa 647 goat anti-rabbit, 1:1000 dilution; Alexa 488 goat anti-mouse IgG (H + L), 1:1000 dilution) solutions in PBS for 2 h.

Imaging was performed with a 20 × 0.75 NA air/60 × 1.3 NA silicon objective lens on a confocal laser scanning microscope (CLSM; Olympus, Fluoview FV3000) or confocal laser scanning microscope (Leica SP8-AOBS) equipped with a 20 × 0.75 NA air objective lens. Z-stack scanning was acquired from the 100 μm depth of constructs at 2–5 μm step sizes, multiple area map scanning was executed to obtain the full image of neurite outgrowth in FLight hydrogel matrix. All images were captured using several excitation wavelengths and the emission signals were captured using suitable detectors (4 GaAsP photomultiplier modules; 405 nm, 488 nm, 561 nm, 640 nm). For example, the 405 nm detector was used to observe nuclei, the 561 nm detector was used for imaging of F-actin or fluorescently labeled microfilaments, and the 640 nm detector was used to image tubulin beta III.

For 3D reconstruction of confocal images, Z-stack scans were first converted to Imaris-readable files using Imaris software (Oxford Instruments, ver. 10.0.0). The 'Surface' function was employed to perform separate 3D reconstructions for different fluorescent signals (e.g., GFP-PODS®, microfilaments). The surface resolution and threshold were set by default, and no filters were applied. The setting was then saved and used for all 3D surface reconstructions. Using the 'Vantage' function, 2D plots were generated. In these plots, the x-axis represented the fluorescent signal volume, while the y-axis indicated the volume ratio of the fluorescent signal to the total 3D scanning volume (n = 3). To calculate the ratio of microchannels in the total 3D hydrogel volume, Z-stack scans of fluorescent-labeled microfilaments were reconstructed using the same method. The percentage of microchannels (P_M_) was determined by the formula:

$$P_{M}=\left(1-\frac{V_{Microfilaments}\;}{V_{Hydrogel}}\right)\times 100\%$$

Similarly, the ratio of PODS® (P_P_) in 3D hydrogel volume was calculated by:

$$P_{P}=\frac{V_{PODS}\;}{V_{Hydrogel}}\times 100\%$$

The total fluorescent intensities of laminin in 3D FLight hydrogel at day 0 and day 4 were measured by Imaris and normalized to hydrogel volume.

### 2.4 Analysis of microstructures and neuronal morphology

To characterize the dimensions of microstructures, the fluorescent Z-stack scans were first transformed into maximum-intensity projection images with the 'Z-project' function in the Fiji software (https://fiji.sc/). To segment microfilaments, the default threshold was applied to all scans. Then, a line perpendicular to microfilaments was drawn using the 'Straight' function. The diameters of both microfilaments and microchannels were measured from the data presented in the 'Plot Profile' panel in Fiji software (n = 3, dataset size = 50).

To assess the alignment of neurites, the acquired tubulin beta III image stacks were initially processed into projections and segmented as previously outlined. Subsequently, the 'OrientationJ' plugin (available at http://bigwww.epfl.ch/demo/orientationj) was used to evaluate the orientation and distribution of the aligned neurites [29]. The structure tensor of the local window was set to 2 pixels and a 'Gaussian' gradient was applied.

For the Sholl analysis of neuronal morphology, the tubulin beta III images were converted to 8-bit images and imported into 'Sholl Analysis 4.0.0′ under the 'Analyze' function. The geometric center of DRG body was determined by masking of DRG body. The step size of Sholl radius was set to be 25 μm and automatic end radius was applied (n = 3). Other parameters such as segmentation, metrics, Sholl decay, and output were chosen by default. The number of intersections at different Sholl radius values was plotted using GraphPad Prism10.0 software.

### 2.5 Measurement of refractive index and light transmission

The refractive indices of the Gel-NB/Gel-SH photoresins containing different amounts of PODS® were measured using an Abbe refractometer (Kern ORT 1RS, KERN & SOHN GmbH). The light transmission (T) of different photoresins was calculated by Beer-Lambert law from optical absorbance (A):

A=2−log(T%)

The optical absorbance was measured using a microplate reader (BioTek Synergy H1 Multimode Reader) from 300 to 450 nm with a 2-nm scan bandwidth.

### 2.6 ELISA for quantification of NGF release from PODS®

PODS®-laden FLight hydrogel samples were incubated in NGF-free Neurobasal medium for 4 days. For NGF-loaded FLight hydrogel samples, NGF was mixed with photoresin at an initial concentration equivalent to the total NGF content in PODS® crystals (8.3 million PODS/mL). At days 1, 2, 3, and 4, 300 μL of culture medium was collected and stored at −20 °C until used. NGF release from PODS® was quantified by Human beta-NGF ELISA kits (R&D Systems DY256) according to manufacturer's protocols.

### 2.7 Mechanical testing

For the compressive tests, cylindrical hydrogel constructs were prepared using the FLight printer with a circular pattern (array of 4 circles), each with a diameter of 5 mm and a height of 4 mm (equivalent to the width of cuvette in the projection direction). The hydrogel samples were tested by unconfined uniaxial compression using Texture Analyzer (Stable Micro systems, TA.XTplus), equipped with a 500-g load cell and a flat plate probe with a diameter of 15 mm. To ensure complete contact between the hydrogel samples and the plates, a preload of 0.1 g was applied. Samples were compressed to a final strain of 30 % at a strain rate of 0.01 mm s^−1^. The elastic compressive modulus was calculated by linear fitting of the initial linear region (0.5–5%) of the stress-strain curve. These tests were repeated four times at 25 °C.

### 2.8 Photorheology

Photorheology analyses were performed on an Anton Paar MCR 302e equipped with a 20 mm parallel plate geometry, 6 mm glass substrate, and OmniCure Series1000 lamp (Lumen Dynamics) used at 60 % output power (equal to 2 mW/cm^2^) with narrow 405 nm bandpass filters (Thorlabs). 80 μL of Gel-NB/Gel-SH photoresins were loaded and left to equilibrate for 3 min at room temperature prior to starting the analysis. Oscillatory measurements were conducted at a shear rate of 2 % and a frequency of 1 Hz, with a gap of 200 μm and 10 s acquisition interval.

### 2.9 Scanning Electron Microscopy (SEM)

Acellular FLight hydrogel samples were printed with different photomasks (or without photomask). The hydrogel constructs were washed several times with mQ H_2_O and fixed in 4 % paraformaldehyde (PFA) solution for 2 h at room temperature. Samples were then washed with mQ H_2_O and dehydrated by stepwise treatment with ethanol in mQ H_2_O (30 %, 50 %, 70 %, 90 %, 100 %) and with Hexamethyldisilazane (HMDS) in ethanol (30 %, 50 %, 70 %, 90 %, 100 %) with a 20-min incubation for each step. After 1 h in 100 % HDMS, the samples were left to dry overnight. Samples were coated with a 10-nm carbon layer (Safematic CCU-010 Carbon Coater). Samples were imaged with SEM (Zeiss Merlin) at an operating voltage of 3–5 kV.

### 2.10 Statistical analysis

Statistical analysis was carried out using GraphPad Prism (x64, v. 10.0.0). Data was analyzed using unpaired *t*-test and presented as mean ± SD unless stated otherwise. Alpha was set to 0.05 and differences between two experimental groups were judged to have statistical significance at ∗*p* < 0.05, where *p* < 0.01, and *p* < 0.001 are represented by “∗∗” and “∗∗∗” respectively; “ns” represents a non-significant difference between groups.

## 3 Results

### 3.1 Stiffness of FLight hydrogel affecting neurite outgrowth

**Fig. 1:**
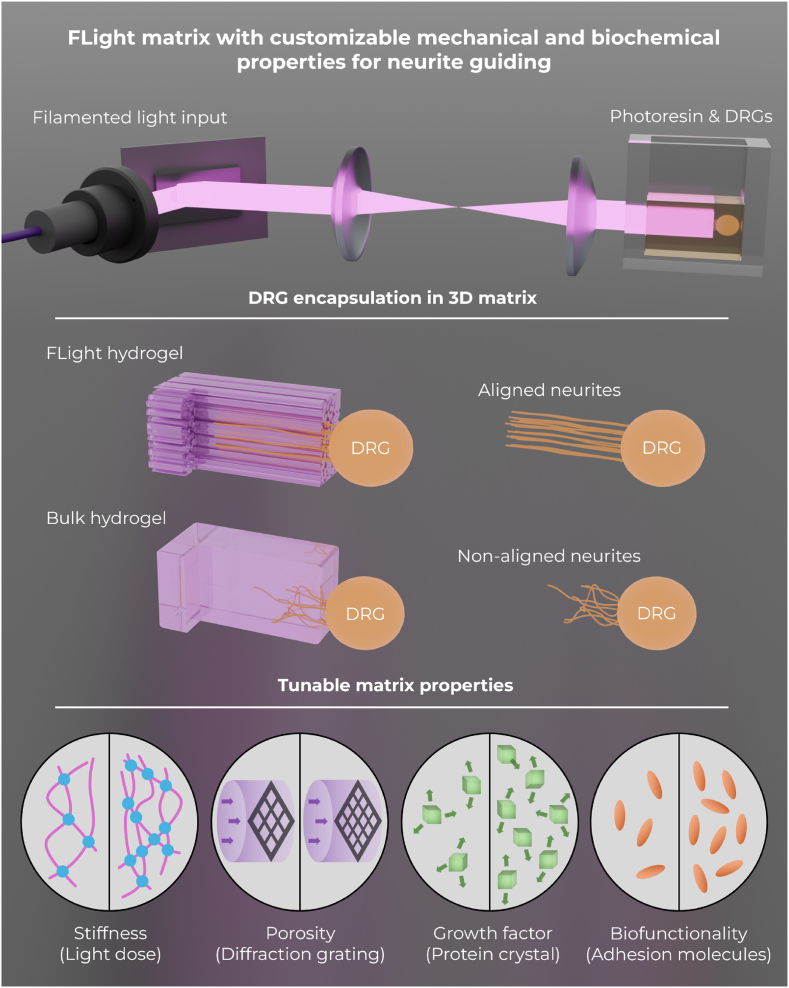
**Schematic illustration of the 3D FLight matrix with tunable properties guiding neurite alignment and growth.** Filamented light projections are generated using a 405-nm diode laser and digital micromirror device (DMD). These shaped light beams are directly projected onto a quartz cuvette containing the photoresin and dorsal root ganglions (DRGs). Through optical modulation instability and self-focusing effects in the non-linear optical media, the intrinsic intensity distribution from the speckle pattern crosslinks the photoresin into hydrogel microfilaments, encapsulating DRGs within the 3D FLight hydrogel constructs. Channel-like void spaces form after removing uncrosslinked photoresin (below the solidification threshold), guiding neurite growth. The properties of the FLight matrices (e.g., stiffness, porosity) are tuned by applying different light doses or using different photomasks. The biochemical properties (e.g., neuron growth factor, bioactive molecules) are adjusted by loading varying concentrations of protein crystal cargo (PODS®) or laminin into the photoresin.

To demonstrate the potential of the FLight hydrogel matrix in guiding axonal outgrowth, freshly harvested chicken DRGs were encapsulated in different 3D matrices with tunable mechanical and biochemical properties (Fig. 1). Due to the excellent reaction kinetics of step-growth polymerization, gelatin-norbornene (Gel-NB) and thiol-functionalized gelatin (Gel-SH) were selected as the bioresin platform. These biomaterials require less light exposure and less radical generation compared to chain-growth polymerization, which ensures a biocompatible printing process for cells [30,31]. A 3 % w/v polymer concentration of Gel-NB and Gel-SH (1:1 M ratio) was used, as it provides an initial hydrogel stiffness range of 0.2–5 kPa, which has also been reported to be optimal for neural tissue engineering and to match the stiffness of native nerve tissues [32,33]. Lithium phenyl-2,4,6-trimethylbenzoylphosphinate (LAP) was incorporated into photoresin formula as photoinitiator (PI) at a final concentration of 0.05 % w/v, allowing photopolymerization of resin at 405 nm wavelengths while maintaining optimal biocompatibility [31].

**Fig. 2:**
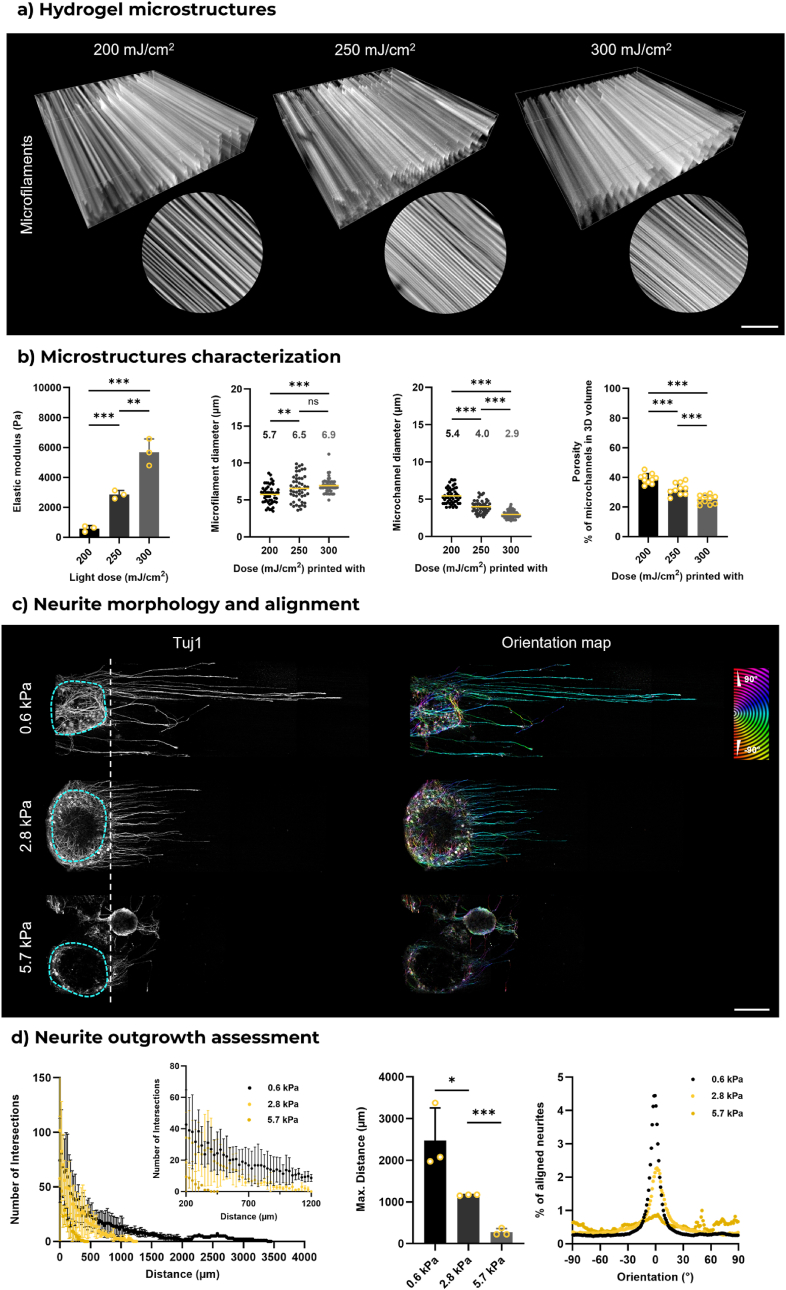
**Matrix stiffness affecting neurite outgrowth in 3D FLight hydrogel. a**) A 3D view of microstructures in different FLight hydrogel constructs printed with varying light doses. Scale bar: 100 μm. **b**) Quantitative analysis of hydrogels and microstructures, including the compressive modulus of hydrogel constructs tested parallel to the direction of microfilaments. Measurements of microstructures include the diameter of microfilaments and microchannels (n = 3, dataset size: 50), and the volume ratio of microchannels in the total 3D hydrogel volume (indicating hydrogel porosity). Numbers depict the mean value of microstructure dimensions. **c**) Representative confocal images of neurite growth in different FLight hydrogels after 4 days of culture. Blue dashed lines indicate the DRG body. The orientation map was generated from Tubulin Beta III (Tuj1) signals. Scale bar: 500 μm. **d**) Sholl analysis and alignment of neurites within FLight matrices with increased stiffness and reduced microchannel size (n = 3). (For interpretation of the references to colour in this figure legend, the reader is referred to the Web version of this article.)

By increasing the light irradiation dose, the elastic modulus of hydrogel and dimension of microstructures can be tuned (Fig. 2a). Specifically, when the projected light dose increased from 200 mJ/cm^2^ to 300 mJ/cm^2^, the elastic modulus showed a notable increase, rising from approximately 0.6 kPa–5.7 kPa (Fig. 2b). However, reducing the light dose below 200 mJ/cm^2^ did not significantly decrease the elastic modulus of the hydrogel constructs (Fig. S1). Additionally, no hydrogel samples could be printed when the light dose fell below 160 mJ/cm^2^, which aligns with the gelation threshold found in photorheological studies. The average diameter of the microfilaments increased from 5.7 μm (200 mJ/cm^2^) to 6.9 μm (300 mJ/cm^2^), while the mean diameter of the microchannels significantly decreased from 5.4 μm to 2.9 μm. This phenomenon may be attributed to the slight crosslinking between microfilaments resulting from the additional light dose. The cumulative dose in areas between the maximum intensity distribution exceeded the crosslinking threshold, leading to changes in the microstructure. Furthermore, the additional crosslinking between microfilaments facilitated a reduced volume ratio of microchannels vs total 3D hydrogel volume, from 40 % to approximately 25 %. However, within the current FLight configuration, it is difficult to decouple the effects of microchannel size and hydrogel stiffness.

To standardize neurite development, DRGs were encapsulated on one side of the hydrogel constructs. This allowed neurites to grow in a single direction, with a maximum outgrowth distance of 4 mm. The assessment of neurite outgrowth was conducted using Sholl analysis, a method for quantifying neurite outgrowth in organoid cultures such as DRGs [34,35]. In this analysis, concentric circles are first labeled and centered on the DRG body. By counting the number of neurite intersections with these circles at different Sholl radius values (25 μm), the longest neurite length and approximate values for the intersection number can be determined. The neurite outgrowth in these matrices suggests that the mechanical properties of FLight matrix mediate neurite development (Fig. 2c and d). Neurite sprouting was significantly reduced in stiffer matrices (5.7 kPa). Sholl analysis revealed a greater number of neurite intersections in the softer matrices (0.6 kPa) at the same Sholl radius. The maximum outgrowth distance decreased from 2.5 mm in soft matrices to 0.3 mm in stiffer matrices by day 4, corresponding to outgrowth rates of approximately 0.6 mm/day and 0.1 mm/day, respectively. Furthermore, limited neurite infiltration within stiff FLight matrices caused many neurites to remain aggregated at the periphery of the DRG body, resulting in improper alignment. These findings underscore the sensitivity of neurite development to the stiffness of 3D matrices and highlight the importance of rational matrix design for nerve tissue engineering applications.

### 3.2 Channel-like structures in FLight hydrogel guiding neurite development

**Fig. 3:**
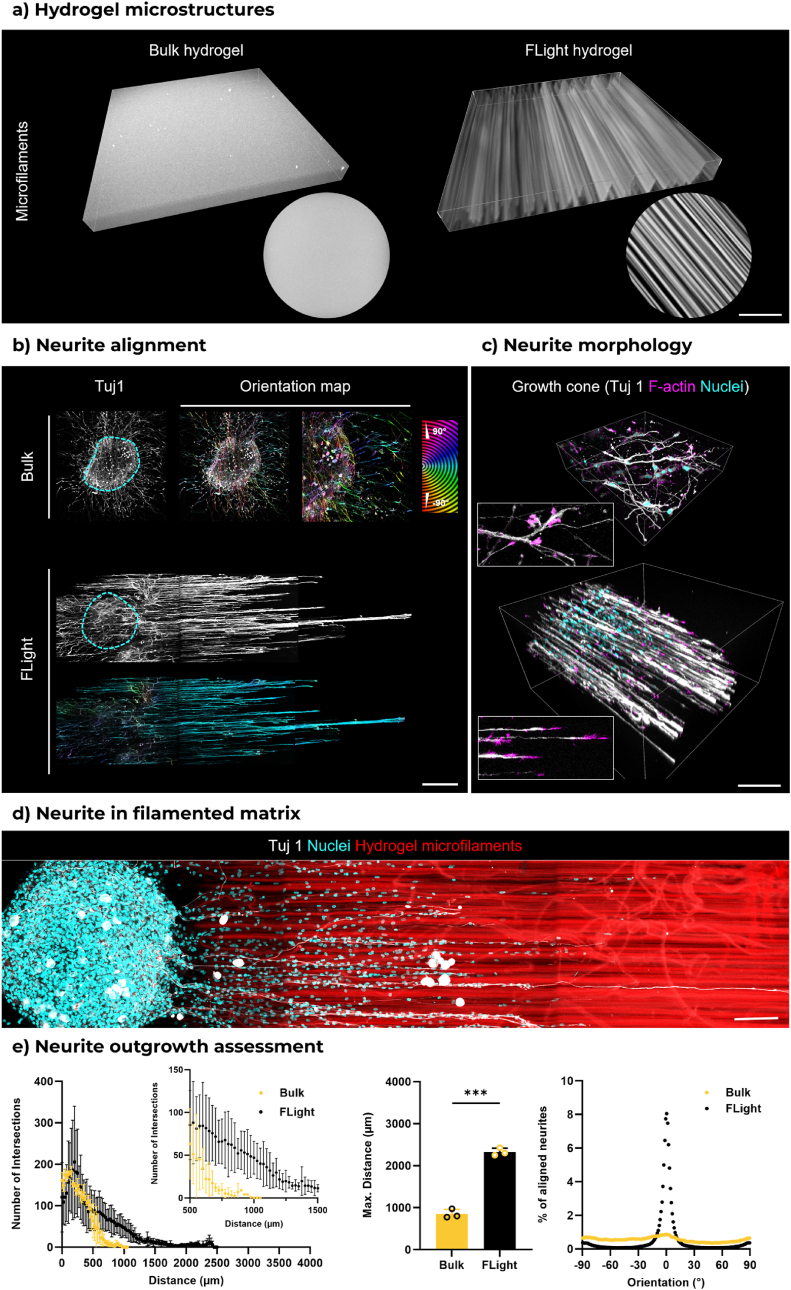
**Anisotropic microchannels in 3D FLight hydrogel guiding neurite outgrowth and alignment. a**) A 3D view of microstructures in bulk and FLight hydrogel constructs printed using fluorescently labeled photoresin. Scale bar: 100 μm. **b**) Representative confocal images of neurite outgrowth in bulk and FLight hydrogel after 4 days of culture. The orientation map was generated from Tubulin Beta III (Tuj1) staining, indicating neurite alignment in the FLight matrix. Scale bar: 500 μm. **c**) Close-ups of neurite morphology in different 3D hydrogel matrices. Zoom-in views highlight growth cones (F-actin – purple). Scale bar: 500 μm. **d**) Zoom-in view of neurite outgrowth in filamented hydrogel matrix and cell infiltration between the fluorescently labeled microfilaments. Scale bar: 100 μm. **e**) Quantification of neurite outgrowth using Sholl analysis (n = 3). Distribution of neurite orientation between bulk and FLight hydrogel. The orientation angle was characterized by the angle deviation from the direction of light projection. (For interpretation of the references to colour in this figure legend, the reader is referred to the Web version of this article.)

To further investigate the role of these channel-like structures in supporting neurite development, the bulk hydrogel matrices, which lack micron-scale pores/voids and topological structures and, therefore, fail to support axonal growth [36,37] were chosen as a comparison. Fluorescently labeled photoresins were successfully crosslinked and formed hydrogel constructs within 4 s (200 mJ/cm^2^) under filamented light projection. Similarly, the bulk hydrogels were fabricated using a light-emitting diode (LED) irradiation with the same light dose. Microfilaments and interconnected microchannels were observed in FLight hydrogel after the removal of uncrosslinked resin and washing, but no microporosities were found in the bulk hydrogel (Fig. 3a). As previously reported [24], the diameter of microchannels was in the range of 3–10 μm, which is comparable to the size of single cells. This ensures cell migration and proliferation while providing effective guiding properties.

All studies on neurite outgrowth were performed after 4 days of culture, as neurites were observed to reach the other end of the hydrogel matrix. The neurites in the FLight matrix grew into the microchannels and aligned highly along the direction of the hydrogel microfilaments (Fig. S2, Video S1), while no alignment of neurites in the bulk matrix was observed (Fig. 3b–d). Furthermore, previous research on neurite growth cone morphology in 2D and 3D collagen matrices revealed a smaller growth cone and a lack of a transition zone in 3D conditions, characterized by the transition from actin to stabilized microtubules [38]. The fewer actin arcs present in the growth cone in 3D matrices allow the extension of microtubules to the leading edge, thus accelerating neurite outgrowth and neuronal polarization [39,40]. The growth cone observed in the FLight matrix exhibited a morphology similar to that reported in previous studies, demonstrating a more dynamic and active growth cone [38,41]. Interestingly, current understandings of nerve tissues suggest that axonal growth is independent of physical interactions with the 3D matrix (e.g., matrix pulling or adhesions) [38]. However, matrix properties such as stiffness, microarchitecture, interconnectivity, and porosity largely determine neural development but are often overlooked [8].

As shown in Fig. 3e, neurites in the FLight matrix exhibited longer average neurite lengths and more intersections at the same Sholl radius value. The maximum outgrowth distances in the bulk and FLight matrices after 4 days of culture were 0.9 mm and 2.4 mm, respectively. A quantitative analysis of neurite orientation revealed that over 86 % of neurites were aligned within a range of −15 to 15° centered on the microfilament direction. In contrast, the percentage of aligned neurites in bulk hydrogels was less than 10 %. These results demonstrate the importance of anisotropic microstructures present in the FLight matrix in supporting neurite outgrowth and guiding its alignment. Given the critical role of neurite outgrowth rate in nerve function repair post-injury [42,43], we further investigated how tunable mechanical and biochemical properties of the FLight matrix potentially accelerate neurite development.

**Fig. 4:**
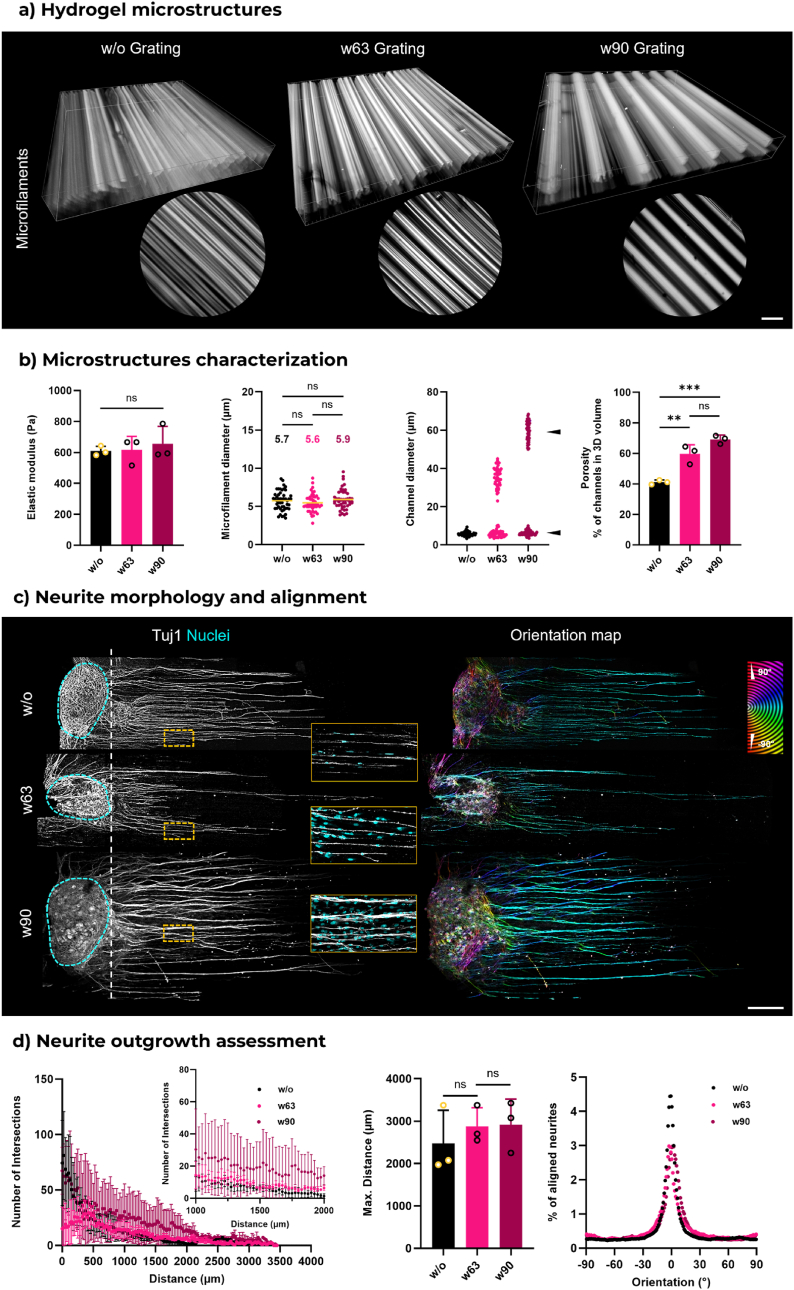
**Macrochannel architecture supporting neurite outgrowth and enhancing cell infiltration in 3D FLight hydrogel. a**) A 3D view of microfilaments in FLight hydrogel constructs printed with different sizes of photomasks. Scale bar: 100 μm. Channel-like void spaces (macrochannels) were created in the FLight hydrogel in addition to microstructures. **b**) Quantification of hydrogel stiffness and evaluation of macro/microstructures in 3D hydrogel with different macrochannels (n = 3, dataset size: 50). Arrows highlight the diameter distribution of the channel-like structures, showing two clusters: microchannels with diameters less than 10 μm and macrochannels with diameters greater than 10 μm. The porosity was determined by the total ratio of channel structures, including macrochannels and microchannels, in the 3D hydrogel volume. Numbers depict the mean value of microstructure dimensions. **c**) Representative confocal images of neurite outgrowth in varying FLight hydrogels. Blue dashed lines highlight the DRG body. Close-ups of neurite-nuclei staining indicate the cell migration in FLight hydrogel. The orientation map was generated from Tubulin Beta III (Tuj1) images. Scale bar: 500 μm. **d**) Sholl analysis and alignment of neurite within FLight matrices with tuned macrochannels (n = 3). (For interpretation of the references to colour in this figure legend, the reader is referred to the Web version of this article.)

Based on these findings, we introduced physical gratings into the optical setup to study the presence of macrochannels. This configuration permitted printing of 3D hydrogels with additional channels without the use of DMD (Fig. 4a). Notably, these additional channels are larger than the microchannels naturally present within the FLight matrix. We hypothesized that larger channels would facilitate the infiltration of supportive cells crucial for neurite outgrowth. To minimize potential alterations to the mechanical properties of the FLight matrix, the hydrogel stiffness was maintained by fixing the projection light dose at 200 mJ/cm^2^. The dose was used in the previous section to print the softest FLight matrix, corresponding to matrix with most neurite outgrowth. The successful printing of hydrogel structures with additional channels (macrochannels) was achieved using metal gratings with widths of 63 μm and 90 μm (w63 and w90) (Fig. 4a–S3a). Although the hydrogel constructs with macrochannels were softer after printing and washing, the microfilament and microchannel structures remained intact. This change was attributed to the fact that there were fewer crosslinked hydrogel portions in the same hydrogel volume, just as no notable changes in hydrogel stiffness were observed when normalized to compression contact area, with a value of about 0.6 kPa. The quantification of the microstructure revealed that there were no significant changes in the mean diameter of the microfilaments, which remained at approximately 6 μm. In comparison to FLight matrices without such macrochannels, the average diameter of micro-channels remained approximately 6 μm in all conditions (Fig. 4b, Fig. S3b). It is noteworthy that the dimensions of the macrochannels exhibited a correlation with the dimensions of the grating utilized to create them. Larger grating dimensions resulted in the formation of macrochannels with greater dimensions. The average diameters of the macrochannels were 35 and 60 μm, respectively. The difference between the width of metal gratings and the diameter of the resulting macrochannels is likely attributable to the Fraunhofer diffraction phenomenon. This effect is particularly pronounced since each metal mesh functions as a highly regular array of apertures. Fig. S3c presents a simulation that illustrates the relationship between the metal grating and the final projected light pattern, also demonstrating that the simulated diameters of the macrochannels closely match the experimentally measured dimensions. Additionally, the macrochannels contributed to an increased void space in the hydrogel volume, resulting in an overall ratio of channel structures that increased from 40 % to 70 %. It is noteworthy that fabrication of even larger macrochannel structures was attempted using gratings with a width exceeding 150 μm. However, the crosslinked hydrogel components were insufficient to maintain the integrity of the entire hydrogel structure.

Our study, then, focused on two sizes of macrochannels and compared them to the basic FLight matrix (i.e., without additional macrochannels). Similar neurite outgrowth was observed under different conditions (Fig. 4c). The Sholl analysis revealed no significant differences in the maximum outgrowth distances across all matrices, which were approximately 2.8 mm, corresponding to an outgrowth rate of 0.7 mm per day (Fig. 4d). No variation in neurite alignment was observed, with over 85 % of neurites aligning along the microfilaments. However, at the same Sholl radius value, more intersections were detected in the FLight matrix with larger macrochannels (w90), particularly at distances greater than 1.5 mm from the DRG body. Additionally, more nuclei were observed between the microfilaments in the macrochannel-containing FLight matrix, as depicted in the yellow inserts in Fig. 4c (also Figure S4). The macrochannels may facilitate cell migration, as they do not require nuclear deformation when cells migrate through them. Overall, more research is needed to understand how these different hierarchical channel-like structures and their sizes affect cell function. However, the FLight approach demonstrated the ability to create these hierarchical microstructures to meet the potential needs of different cells.

### 3.3 Biochemical cues enhance the guiding properties of FLight hydrogel for neurite development

It is well established that growth factors in the microenvironment play a crucial role in nerve development and repair, particularly in guiding axon regeneration after spinal cord injuries, by supporting neuronal survival and stabilizing axons [44]. Among the various growth factors, NGF is a primary mediator of the repair of both central and peripheral nervous systems, such as nociceptive axons that sense pain [45,46]. Localized application or controlled release of NGF in the microenvironment can guide regenerating axons through injury sites, re-establishing connections, and innervating target tissues. High concentrations of NGF or gradients within the hydrogel matrix have been reported to be highly effective in attracting axon growth [47]. The combination of growth factors with other complementary strategies, such as topological cues in scaffolds, has become a promising approach in neural tissue engineering for bridging nerves [37].

**Fig. 5:**
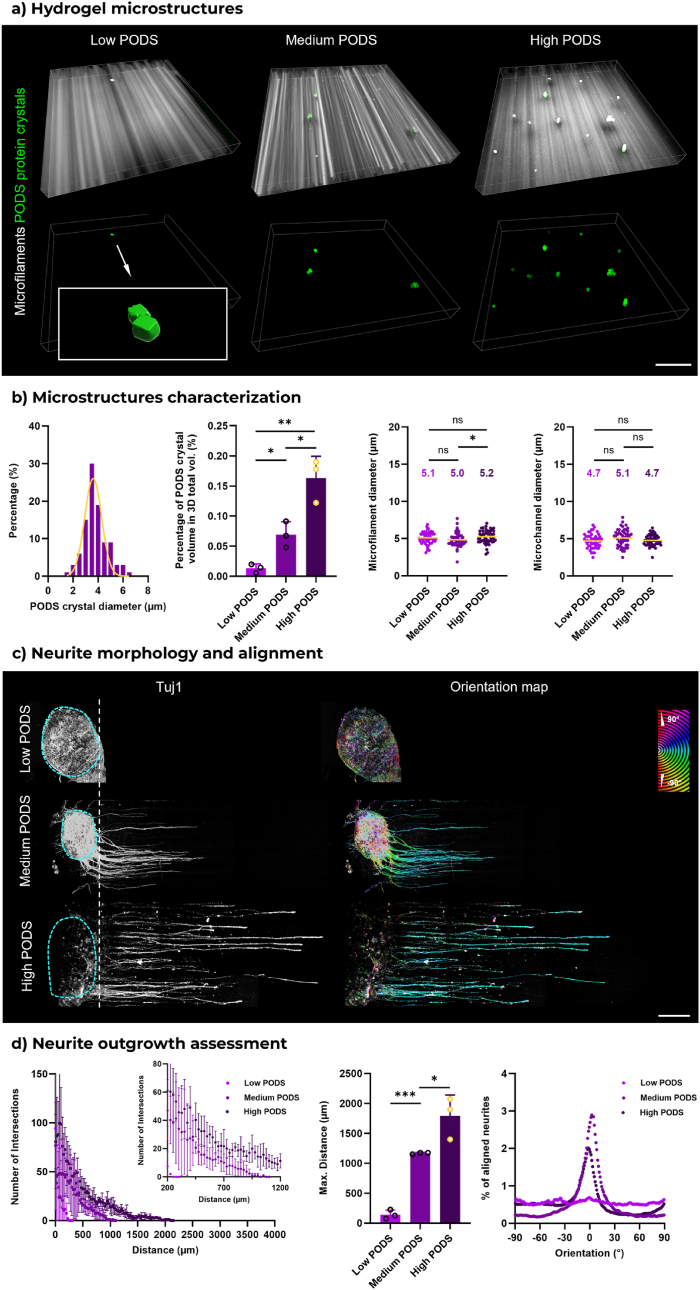
**Locally-released growth factor supporting neurite growth in 3D FLight hydrogel. a**) A 3D view of microfilaments and fluorescently labeled protein crystals (GFP-PODS) in FLight hydrogel constructs with different concentrations. Low: 0.33 million PODS/mL; Medium: 1.67 million PODS/mL; High: 8.3 million PODS/mL. Different amounts of cubic PODS microcrystals were homogeneously mixed with photoresin and encapsulated within the FLight matrix to release nerve growth factor (NGF) during culture. Scale bar: 50 μm. **b**) Quantification of the dimensions of microparticles and microstructures in 3D FLight hydrogel (n = 3, dataset size: 50). Numbers depict the mean value of microstructure dimensions. **c**) Representative confocal images of neurite growth in FLight hydrogels loaded with varying concentrations of NGF-PODS. Blue dashed lines indicate the DRG body. The orientation map was generated from Tubulin Beta III (Tuj1) images. Scale bar: 500 μm. **d**) Sholl analysis and alignment of neurites within PODS-laden FLight matrices (n = 3). (For interpretation of the references to colour in this figure legend, the reader is referred to the Web version of this article.)

Here, we introduce a protein crystal sustained-release system, PODS®, which allows for the local release of NGF into the 3D FLight matrix. PODS are polyhedral protein assemblies forming microcrystals with cubic structures [48,49]. The active protein cargo, such as NGF, is released to the microenvironment when cells secrete proteases to degrade PODS [50]. When mixing fluorescently labeled PODS (GFP-PODS) at different concentrations with photoresin to print 3D FLight matrices (Fig. 5a–S5a), it was observed that PODS were encapsulated within matrices. Their distribution within the resin could be more homogenous if sonication or more advanced mixing techniques were used. The diameter distribution of PODS revealed an average diameter of 4 μm (Fig. 5b). Given that PODS size is within the same range as microstructures, the potential scattering caused by these cubes on the formation of microstructures is noteworthy. However, there was no significant difference detected in the refractive index (RI) of photoresin mixed with different PODS concentrations compared to photoresin without PODS (Figure S5b-c). Although light transmittance slightly decreased with increasing PODS concentration, even at the highest concentration, light transmittance at a wavelength of 405 nm remained above 80 %. Moreover, the proportion of PODS microcrystals in the overall hydrogel volume increased with higher amounts of PODS, but the volume ratio was below 0.3 % v/v, which is negligible for the FLight printing process. Therefore, it can be concluded that the direct disruption of self-focusing effect and defect of microstructures was unlikely to happen. The quantification of microstructures corroborated this conclusion. The diameter of the microfilaments and microchannels exhibited comparable distributions at all PODS concentrations, with an average diameter of approximately 5 μm.

Subsequently, the DRGs were encapsulated together with NGF-PODS at three different concentrations within FLight matrix and cultured without the addition of NGF to the medium (Low: 0.33 million PODS/mL; Medium: 1.67 million PODS/mL; High: 8.3 million PODS/mL). Surprisingly, significant differences in neurite development were observed after four days of culture (Fig. 5c). In the 3D matrix encapsulating the lower concentration of NGF-PODS (0.33 million PODS/mL), neurites exhibited minimal outgrowth. The Sholl analysis of neurites once again confirmed the positive effects of 10.13039/100017798NGF in supporting neurite development (in terms of the number of intersections) and neurite outgrowth distances (Fig. 5d). The maximum outgrowth distances were found to be 0.2 mm at the low concentration of NGF-PODS. For comparison, neurites from DRG cultured in hydrogel matrices without NGF supplementation from either PODS or culture medium exhibited minimal outgrowth (i.e., control group, Fig. S6), with no significant difference in neurite extension observed between the control group and samples containing low concentrations of NGF-PODS. However, a maximum neurite outgrowth distance of 1.8 mm was confirmed in the condition with high concentrations of NGF-PODS. The maximum outgrowth distance achieved is comparable to that observed when growth factor was added to the culture medium, demonstrating that these NGF-PODS can serve as an alternative source of exogenous growth factor. The kinetics of NGF release from PODS at varying encapsulation concentrations were quantified (Fig. S5d). The highest NGF concentration in culture medium was detected from 3D matrices encapsulated with the highest concentration of NGF-PODS (i.e., 8.3 million PODS/mL). A control sample, where the same NGF amount as the highest NGF-PODS concentration was directly mixed with photoresin, showed a marked decrease in NGF concentration over time compared to high concentration of NGF-PODS. Notably, neurites remained aligned in the 3D matrix under medium NGF-PODS conditions, despite the slower neurite development. This further highlights the necessity of integrating topological guidance cues with biochemical cues within the microenvironment.

### 3.4 Modification of FLight matrix with bioactive substrate improves neurite development

In addition to the mechanical properties of the FLight matrix and exogenous growth factors, the presence of bioactive molecules in a 3D matrix is essential. This is evident from the complex chemical composition of the neural ECM. These molecules, such as laminin (LN), fibronectin (FN), and collagen type IV (Col IV), interact with signaling receptors on cell surfaces and adhesion molecules to promote neural tissue repair [51,52]. Although FN and Col IV are also important, LN has been demonstrated to be superior in promoting remyelination *in vivo* after peripheral nerve injury. This may be due to LN-binding to Schwann cell surface receptors, which aids cell survival and modulates downstream gene expression (e.g., upregulation of myelin-specific proteins) to create an optimal microenvironment for myelin regeneration [53]. Therefore, LN, FN and their mixture were employed as proof-of-concept bioactive molecules, which were loaded onto FLight matrices with the objective of enhancing neural development.

**Fig. 6:**
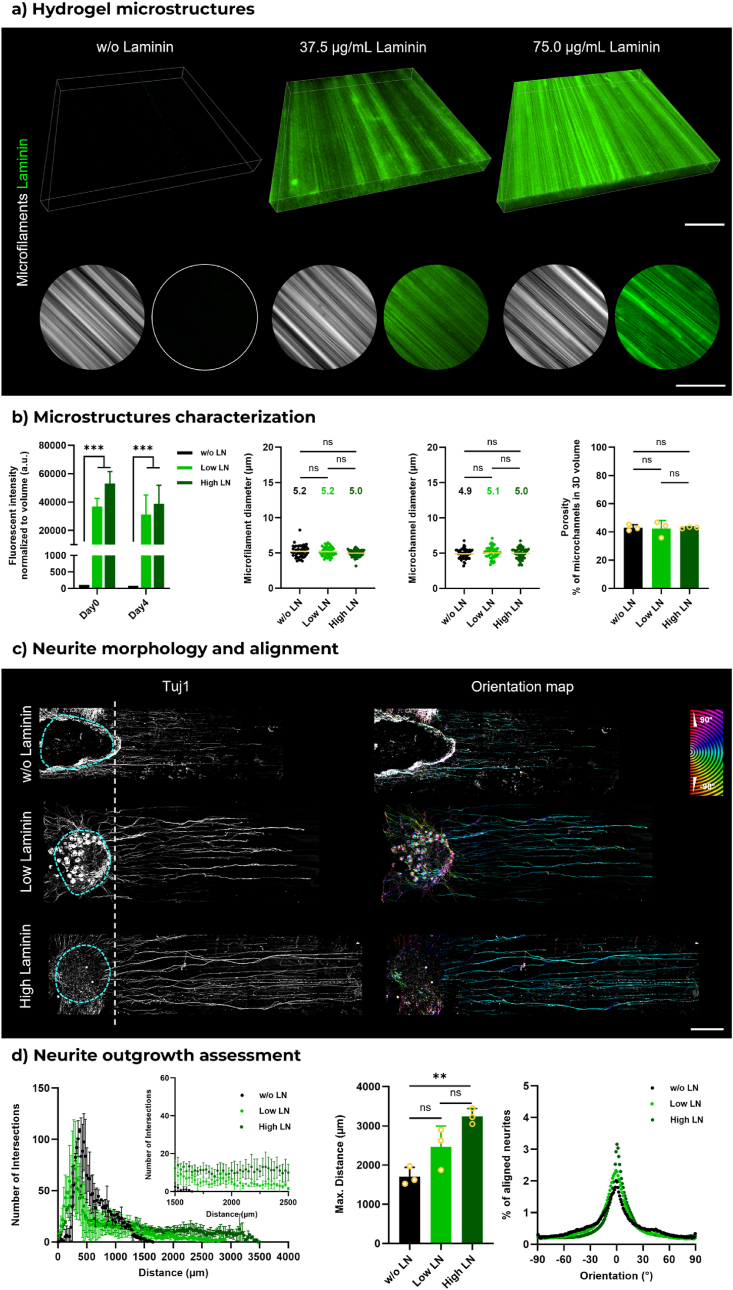
**Bioactive molecule supporting neurite outgrowth in 3D FLight hydrogel. a**) Confocal images of laminin (LN) and microfilaments in 3D FLight matrices printed with different concentrations of LN. Low: 37.5 μg/mL; High: 75 μg/mL of LN in photoresins. Scale bar: 50 μm. **b**) Normalized fluorescent intensity of LN from confocal microscopic images (left), and quantification of the dimensions of microstructures in 3D FLight hydrogel (n = 3, dataset size: 50). Numbers depict the mean value of microstructure dimensions. **c**) Representative confocal images of neurite growth in FLight hydrogels loaded with varying concentrations of LN. Blue dashed lines indicate the DRG body. The orientation map was generated from Tubulin Beta III (Tuj1) images. Scale bar: 500 μm. **d**) Sholl analysis and alignment of neurite within LN-laden FLight matrices (n = 3). (For interpretation of the references to colour in this figure legend, the reader is referred to the Web version of this article.)

To maintain the native biochemical properties of LN, unmodified LN was mixed and printed with the photoresin at different concentrations. The resulting FLight matrix was then subjected to immunofluorescent staining to assess the loading of LN. The control group exhibited no discernible fluorescent signals (Fig. 6a). Nevertheless, fluorescent signals were observed in the FLight matrix encapsulating LN, with increased fluorescent intensity in matrices encapsulating a higher concentration of LN. The fluorescent signals exhibited an overlap with the hydrogel microfilaments, thereby confirming the successful loading of LN into the hydrogel microfilaments. The impact on the enhancement of cellular functions of encapsulating LN within a hydrogel matrix through simple mixing has also been demonstrated recently [54]. Moreover, similar intensity signals to day 0 were detected after four days of culture, indicating the stability of the LN loading process (Fig. 6b–S7). Furthermore, no significant changes in the dimensions of the microstructures (∼5 μm for microfilaments and microchannels) and porosity were observed in the FLight matrix encapsulating LN.

Upon evaluating neurite outgrowth within LN-laden FLight matrices, a notable enhancement in neurite development was observed in hydrogel constructs containing high concentrations of LN (Fig. 6c). The Sholl analysis demonstrated that the maximum outgrowth distance of neurites in the 3D matrix loaded with a high concentration of LN was longer than that observed in the control group. The maximum distances were 3.2 mm (high LN) and 1.7 mm (w/o LN), corresponding to maximum outgrowth rates of approximately 0.8 mm per day and 0.4 mm per day, respectively (Fig. 6d). Furthermore, more intersections were observed at locations distant from the DRG body (particularly when Sholl radius >1.5 mm) in LN-laden matrices. However, no differences were observed in neurite alignment, with ∼80 % of neurites aligned along the direction of microfilaments under all three conditions. Given the positive role of other bioactive molecules like FN in neurite outgrowth, additional filamented hydrogel matrices were prepared by adding FN or a mixture of FN and LN (FN-LN) to the photoresins (Fig. S8). The concentration of FN was 75 μg/mL, equivalent to the protein concentration in high LN condition. FN-LN mixture (1:1) was added at a total concentration of 75 μg/mL to the photoresin. After FLight projection and 4 days of incubation, the presence of different molecules was confirmed via immunofluorescence staining. The average diameter of microfilaments and microchannels in various hydrogel samples were measured to be ∼5 μm, and no significant differences in porosity was found (Fig. S8b). Compared to the FN and FN-LN groups, the encapsulated DRGs in LN-laden matrices exhibited the longest neurite outgrowth distance. However, more intersections were observed in FN-LN loaded FLight scaffolds in the Sholl radius of 1.5–2.5 mm. Highly aligned neurites were observed in all conditions.

## 4 Discussion

FLight technology offers a rapid, flexible, and scalable approach for the fabrication of hydrogel matrices consisting of highly aligned microfilaments, which have relevance towards regeneration of nerve injuries or neuronal tissue engineering. The overview of the maximum neurite outgrowth distance highlights multiple critical factors (Fig. S9), illustrating that FLight hydrogels, as tunable scaffold systems, can be adjusted to optimize matrix properties to further support neurite development. In the first part of this work, we demonstrated the tunability of the matrices’ mechanical properties by adjusting the light intensity and incorporating diffraction gratings between the filamented light beam. In the second part, we studied how incorporating growth factors or biological molecules into the photoresin can enhance neurite outgrowth.

The critical effects of ECM stiffness and porosity on cell migration and proliferation in 3D microenvironments have been extensively studied with various cell types. Stiffer hydrogel matrices or less porosity significantly restrict cell migration, especially when the size of void spaces is smaller than the nuclei, due to its limited deformability [55]. These factors together act as physical cues present within the hydrogel matrix and play a crucial role in nerve growth [56]. Therefore, a rational design of matrix stiffness of FLight constructs is critical to support neurite outgrowth [8,37]. Increasing the light intensity (200–300 mJ/cm^2^) allowed an increase in stiffness (0.6 kPa–5.7 kPa), but this stiffness increase was accompanied by an increased matrix crosslinking and narrowing of microchannels (from ∼5.4 μm to 2.9 μm). Here, we observed the longest neurite outgrowth in the softest matrix (∼0.6 kPa) and a significant restriction on neurite development in the stiffer FLight matrix. This is consistent with current literature, softer 3D hydrogels generally enhance neurite outgrowth, as demonstrated by Lampe et al., who observed the longest outgrowth in the softest gel (0.5 kPa) [57]. Besides, recent *in vitro* studies on the engineering of fiber-scaffolding/microgel for neurite guiding have reported a maximum neurite outgrowth rate of 0.2–0.6 mm per day [14,19,47,58,59], which is comparable to the observation of neurite development in the FLight soft matrix (0.6 mm per day). However, in our study, it is challenging to distinguish whether the reduced neurite outgrowth in stiffer FLight constructs is due to the increased stiffness or the reduced dimension of microchannels. In our future studies, we intend to modulate the speckle patterns of the incident light and self-focusing effect to maintain constant dimensions of microarchitectures (e.g., a microchannel diameter of 5.4 μm) while varying matrix stiffness to observe the effects on neurite development.

In the subsequent experiment, we fixed the matrix stiffness (∼0.6 kPa) but added meshes as diffraction gratings to impart larger macrochannels (>40 μm) within the constructs in addition to the microchannels (∼5.4 μm) generated through FLight. In this case, we did not observe changes in the neurite outgrowth between the groups containing the larger macrochannels, but yes, on the number of cells infiltrating. For instance, Omidinia-Anarkoli et al. reported that neurite alignment is optimal in voids between 10 and 30 μm, with larger voids leading to multidirectional growth [60]. In contrast, our study demonstrated neurite alignment even in larger channels, likely due to the presence of microfilaments on the inner surfaces of the macrochannels, which serve as topological cues for guiding neurite growth. Interestingly, previous studies have demonstrated the pivotal contribution of Schwann cells and endothelial cells in the nerve repair processes [61,62]. These cells require adequate porosity for migration and proliferation during tissue regeneration [55,63]. Therefore, we believe that the presence of microchannels might not contribute directly to neurite outgrowth but to the migration of supporting cells, such as Schwann cells, endothelial cells, macrophages, fibroblasts. Additionally, further investigation is necessary on the impact of different hierarchical channel structures in FLight matrices on cell-cell interactions and nerve repair. For example, it would be beneficial to determine how different cells are influenced by varying levels of microstructures (microchannels vs. macrochannels) and thereby enhance nerve development. For effective bridging of nerve defects, for instance, infiltration of glial cells (e.g., Schwann cells) is important for myelination of the outgrowing axons and promoting signal transduction [61]. For larger defects (discussed below), the generation of neovasculature from endothelial cells is important to support the nutritional demands of the other cells (neurons, glial cells, etc.) [59].

As previously highlighted, the bridging of nerve defects is driven by the support of a multitude of cells, where the glial cell infiltration into the matrices is essential for chemotactic guidance of the axons (i.e., secretion of growth factors from the cells) and later myelination for improved signal transduction. Neovasculature through endothelial cell infiltration is essential for supporting the nutrient transport to the newly growing cells. These aspects are essential for rapid nerve bridging and to counteract the Wallerian degeneration of the nerves in the distal region [5]. While the infiltration of Schwann cells is a transient process occurring over several weeks, PODS can act as a surrogate and release essential growth factors for neurite guidance. In this work, we demonstrated that the local release of NGF from PODS-encapsulated FLight matrix reduces the need for additional NGF in the culture dish. This strategy could be a robust approach for the localization of growth factor release from the grafts. Furthermore, PODS are also available in a variety of formulations that encapsulate other essential growth factors, such as vascular endothelial growth factor VEGF, which play a crucial role in bridging larger defects through neovascularization. In addition to different types of PODS encapsulated in the FLight matrices, the introduction of macrochannels (i.e., through diffraction gratings) can further aid the formation of larger arterioles and venules to support the nutritional demands of the regenerating tissue.

Our preliminary results also indicate the positive effect of bioactive molecules on neurite growth when combined with the topological cues present in the FLight matrix. To further improve the long-term stability of bioactive molecules, they could be enzymatically crosslinked using microbial transglutaminase (mTG) [64]. Similar to previous reports, LN facilitated the longest neurite outgrowth distance compared to FN [65]. However, the combination of FN and LN in FLight hydrogels appeared to promote more neurites outgrowth from DRG bodies, as evidenced by the observation of more intersections. Considering the complex composition of bioactive molecules in the hierarchical structures of neural tissues, using decellularized extracellular matrix (dECM) [66] is another promising strategy for creating nerve scaffolds or engineering *in vitro* tissue models of various types. Relevant studies have revealed that these dECM are rich in matrix components similar to the composition of native tissues, which are effective in supporting the cellular functions of various cell types. Recent studies on light-based bioprinting technologies have demonstrated the possibility of printing 3D hydrogel constructs through crosslinking of tyrosine groups in dECM using ruthenium (Ru-SPS) as a photoinitiator under visible light [67,68].

Furthermore, regarding clinical applications such as tissue grafts to replace autografts for nerve repairs, the current size of the FLight construct is too short to treat large defects (>30 mm in hands and legs, and >10 mm in fingers) [5]. Our current approach of sideways FLight projection within cuvettes is limited to a few millimeters of cuvette width due to light scattering and attenuation as it travels through the resin, which is further dependent on the photoresin concentration and composition [69]. In the present study, the attenuation coefficient of the LAP photoinitiator is quite low (0.25 cm^−1^), allowing for approximately 90 % of the incident intensity to exit the 4-mm cuvette. Consequently, the matrix stiffness is constant along the length of the constructs. However, for longer constructs and for highly attenuating resins (i.e., riboflavin-based photoinitiation systems) [31], we may observe a higher change in the matrix stiffness throughout the length, which can affect the neurite development through the matrices. To circumvent this, we are developing a top-down continuous printing apparatus, where structured light is projected onto the air-resin interface while simultaneously feeding photoresin into the cuvette. This approach removes the size limitations imposed by the cuvette and photoresin. Photoabsorbers can be employed to prevent light penetration into the already-crosslinked layers and to prevent overcrosslinking of the constructs [70].

The presence of connective tissue between the fascicular arrangement of myelinated axons within nerve tissues allows for enhanced strength and stretchability. Consequently, nerve autografts (e.g., sural nerves) or decellularized nerve grafts possess the necessary mechanical properties, which facilitate their handling and anastomosis at the defect site. This aspect is challenging with current soft FLight matrices (∼0.6 kPa modulus), which can easily break when compressive forces (e.g., during handling through forceps) or shear forces (e.g., during suturing) are applied to these hydrogels. One potential strategy for providing the necessary mechanical support and stability, while permitting the relevant axonal guidance, is to combine other materials or biofabrication technologies that can produce a stiffer outer shell (e.g., melt electrowriting) [71] with the softer core produced with FLight. In this instance, a core-shell structure comprising a softer core allows for cell infiltration, while a stiffer shell enables improved handleability and suturability. Furthermore, future studies will also evaluate the electrophysiology of the DRG within the flight matrices to understand the potential as NGC scaffolds.

The preceding discussion briefly highlights the potential of FLight matrices for tissue repair grafts, yet this technology is equally applicable to *in vitro* nerve tissue modeling. For example, the FLight approach provides an engineered matrix platform for the study of the effects of 3D matrix stiffness on the guidance and migration of different cell types (glial cells, fibroblasts, etc.), as well as of the potential effects of mechanical confinement on the mechanotransduction in the cells [72]. Engineered tissue models are also contributing to the 3R initiative in animal research, which aims to reduce, refine, and replace animal testing. Notably, up to 50 % of pre-clinical animal studies are non-reproducible [73], and over 90 % of drugs deemed pre-clinically safe and effective fail in Phase III trials. The development of a patient-specific tissue-screening model represents a significant step forward in the creation of an efficient and precise testing environment. Recent advances in the transdifferentiating of patient-derived fibroblasts (iPSC) to tissue-specific cells have enabled the development of disease models that assess inflammatory responses and determine patient-specific safe doses [74]. Furthermore, FLight approach is also capable of multi-material printing, as with the hydrogel interface demonstrated in our previous studies [24]. This approach is promising for engineering tissue interfaces such as neuromuscular junctions (NMJs). In these tissues, the PODS approach is particularly beneficial when a gradient of distinct growth factors is necessary for such co-culture systems. For example, TGF-beta and NGF have been demonstrated to enhance the maturation of muscle and nerve tissues respectively, thus are widely used in the field of tissue engineering. An appropriate gradient of these growth factors also facilitates the formation of NMJs. Nevertheless, the conventional method of adding growth factors uniformly to the culture medium presents a significant challenge to achieving this gradient [75,76]. Similarly, the successful engineering of peripheral nerve injury models with fascial tissue structures using local biochemical cues has the potential to provide insights into the inflammatory and regenerative responses of neural tissues and helping to identify potential therapeutic targets [77]. We believe that the FLight technology will be a pivotal biofabrication tool for a broad spectrum of aligned tissues and their interfaces.

## 5 Conclusion

In this study, we successfully developed filamented hydrogels to support neurite development in the 3D matrix. Our studies demonstrated that FLight technology, beyond cell alignment, is a flexible platform that allows for the modulation of the hydrogels’ 3D inner structure with tunable properties regarding matrix stiffness, pore size, protein embedding and matrix composition. This technology can be a valuable tool for elucidating the mechanobiological mechanisms underlying anisotropic cell growth and as a fabrication platform for a potential scaffold alternative for nerve repair.

## CRediT authorship contribution statement

**Hao Liu:** Writing – review & editing, Writing – original draft, Visualization, Validation, Project administration, Methodology, Formal analysis, Data curation, Conceptualization. **Anna Puiggalí-Jou:** Writing – review & editing, Writing – original draft, Visualization, Validation, Methodology, Investigation, Formal analysis, Data curation, Conceptualization. **Parth Chansoria:** Writing – review & editing, Methodology, Investigation. **Jakub Janiak:** Writing – review & editing, Methodology, Investigation. **Marcy Zenobi-Wong:** Writing – review & editing, Supervision, Funding acquisition, Conceptualization.

## Declaration of competing interest

The authors declare that they have no known competing financial interests or personal relationships that could have appeared to influence the work reported in this paper.

## Data Availability

The data that support the findings of this study are openly available at ETH Zurich Research Collection at https://doi.org/10.3929/ethz-b-000682496.
