# Supplementary material for: Filamented hydrogels as tunable conduits for guiding neurite outgrowth
Source: Mater Today Bio. 2025 Jan 11;31:101471. doi: 10.1016/j.mtbio.2025.101471 (PMC11787030; doi:10.1016/j.mtbio.2025.101471)
Supplement: Multimedia component 1 [file mmc1.docx]

Supplementary information for

**Filamented Hydrogels as Tunable Conduits for Guiding Neurite Outgrowth**

**Hao Liu^#^, Anna Puiggali-Jou ^#^, Parth Chansoria, Jakub Janiak, Marcy Zenobi-Wong***

Content

Figure S1 – S9

Video S1


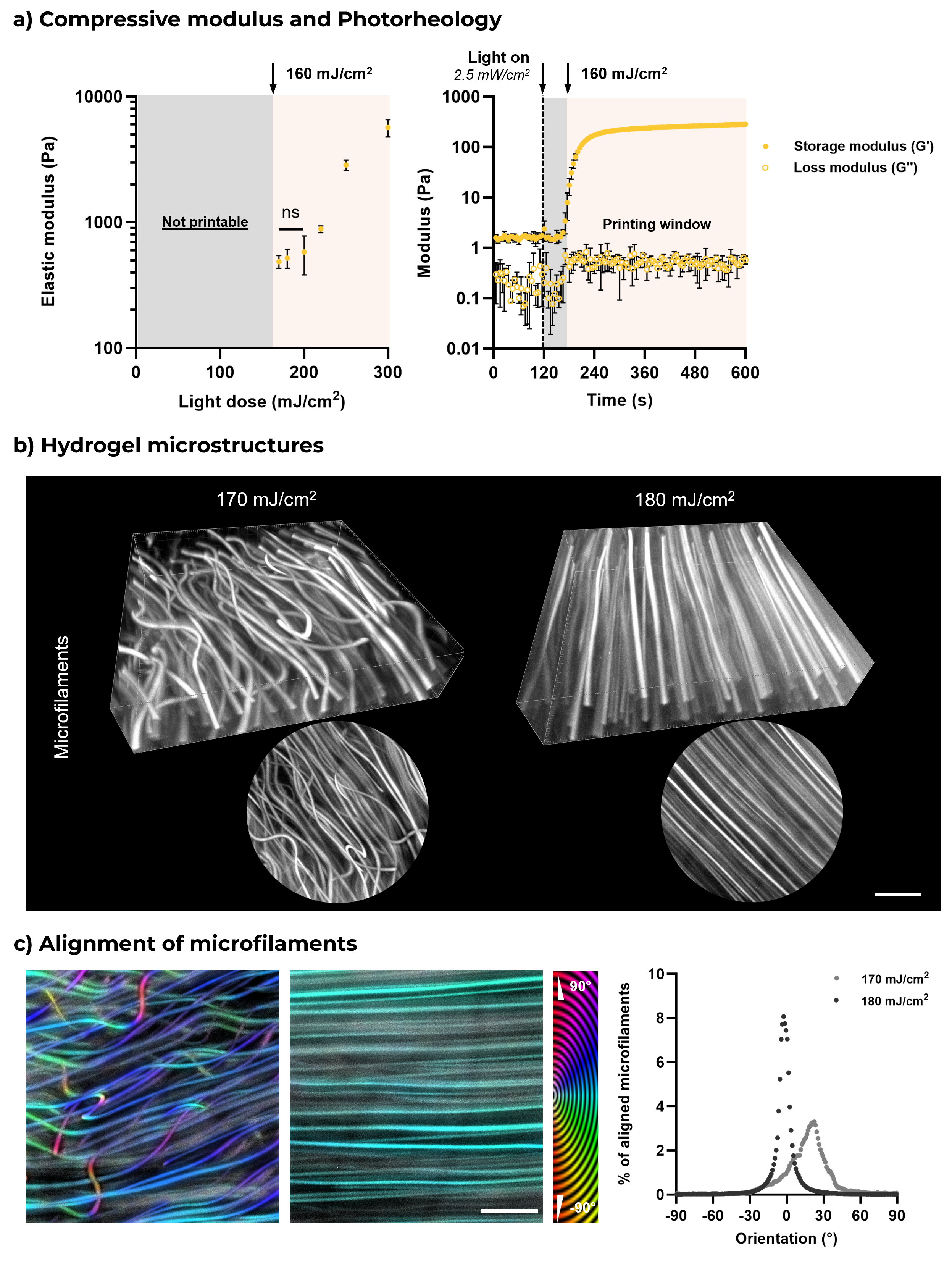


**Figure S1.** **Light dose and matrix stiffness.** **a**) Compressive modulus of hydrogel constructs printed with varying light doses (left), and photocrosslinking kinetics measured by a photorheometer (right). The arrow marks the photocrosslinking threshold; hydrogel constructs could not be printed at light doses below 160 mJ/cm^2^. **b**) A 3D view of microstructures in FLight hydrogel printed with light doses of 170 mJ/cm^2^ and 180 mJ/cm^2^. Microfilaments in the hydrogel samples printed with light dose of 170 mJ/cm^2^ exhibit a misaligned morphology at the microscopic level after washing, leading to poor handling properties. Scale bar: 100 µm. **c**) Orientation maps and assessment of microfilament alignment in various hydrogel samples. Scale bar: 100 µm.


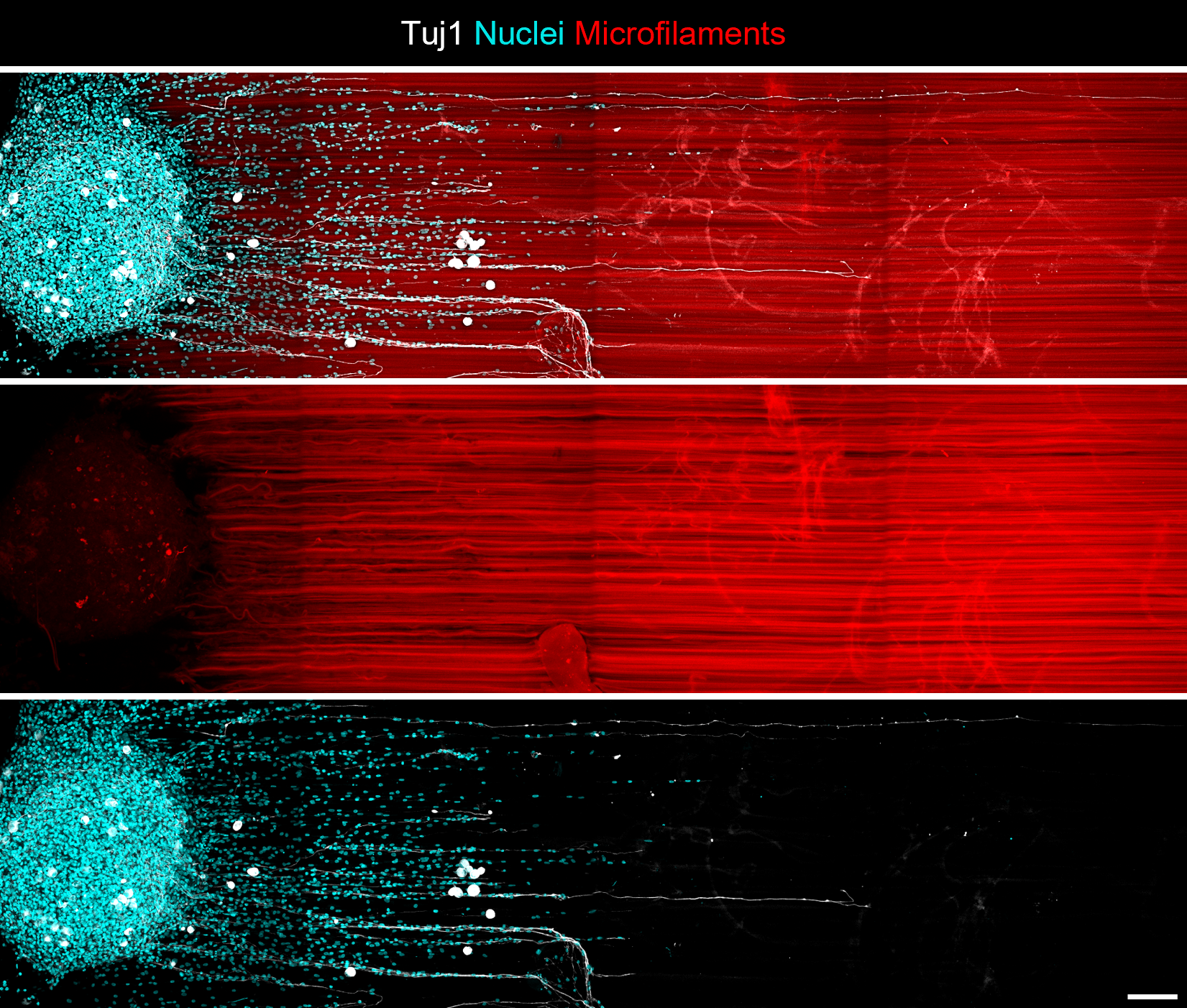


**Figure S2.** **Neurites’ outgrowth aligned along hydrogel microfilaments.** A maximum-intensity-projection image from confocal scanning shows neurites aligned within the FLight hydrogel matrix. The hydrogel microfilaments are fluorescently labeled, highlighting their structure and interaction with the neurites. Scale bar: 100 µm.


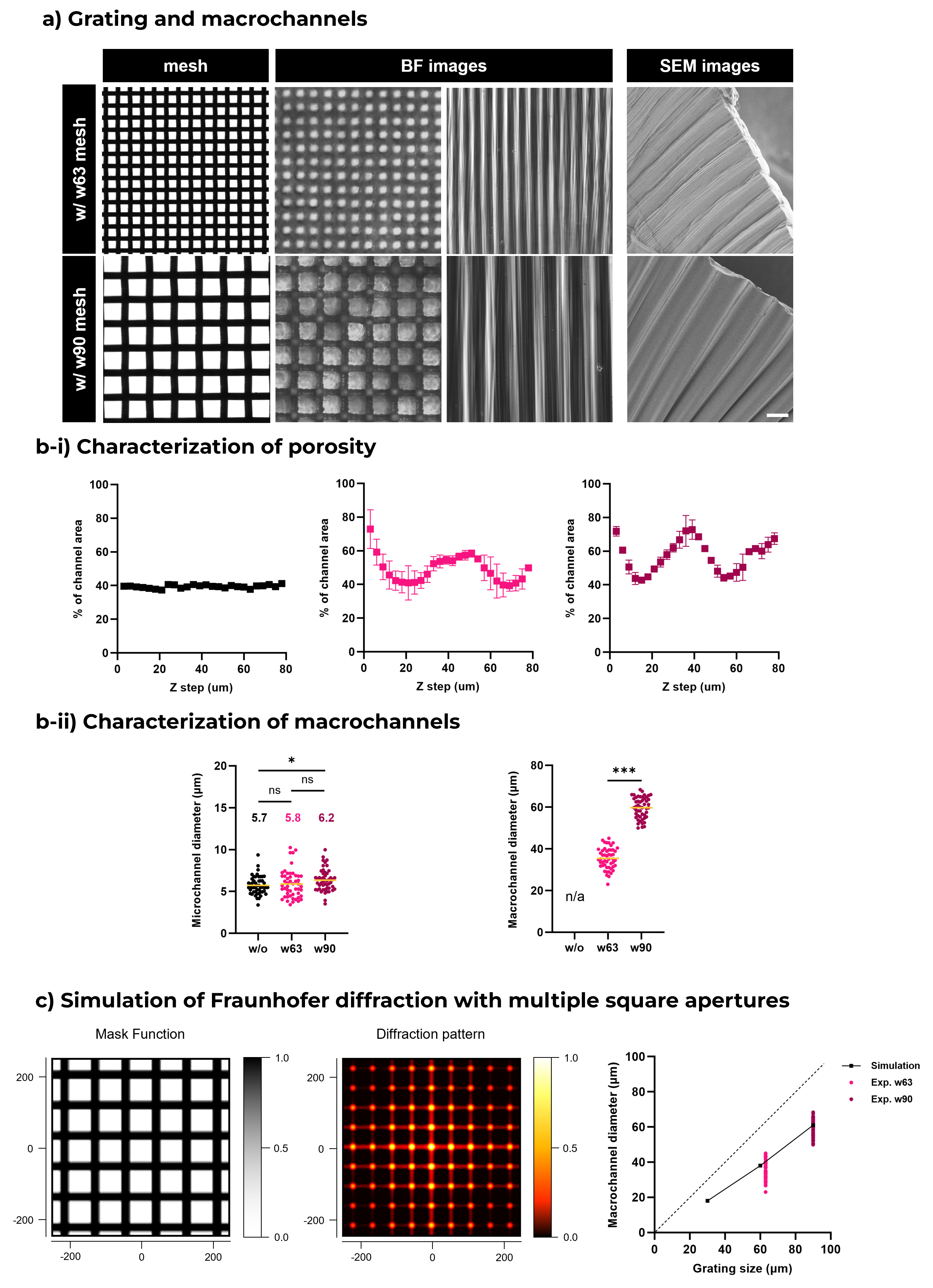


**Figure S3. Diffraction grating in FLight setup and macrochannel structures in FLight hydrogel matrices.** **a**) Bright-field images of diffraction gratings with different widths (left panel), bright-field images of macrochannels/microfilaments in a cross-sectional and side view (middle panel) and scanning electron microscopy images of hydrogel constructs (right panel). Scale bar: 50 µm. **b**) Characterization of porosity at different z-steps (i) and dimensions of microchannels/macrochannels in FLight matrices printed using diffraction gratings. **c**) Simulation of Fraunhofer diffraction in FLight systems. Left: Representation of a metal mesh mask function in the FLight setup, consisting of multiple square apertures each 60 µm wide, with coordinate units in micrometers for x-y axes. Middle: Diffraction pattern displayed with a color bar that indicates relative light intensity values at various positions of the light projection pattern, using a mesh width of 60 µm (units: µm for x-y coordinates). Right: Correlation between the simulated grating size (i.e., mesh size) and the dimensions of the printed macrochannels, accompanied by a comparison with experimental values. The dotted line represents the relationship between mesh width and printed macrochannel diameter under ideal conditions, without considering Fraunhofer diffraction. The Fraunhofer diffraction simulation was conducted as outlined in the previous report[1].


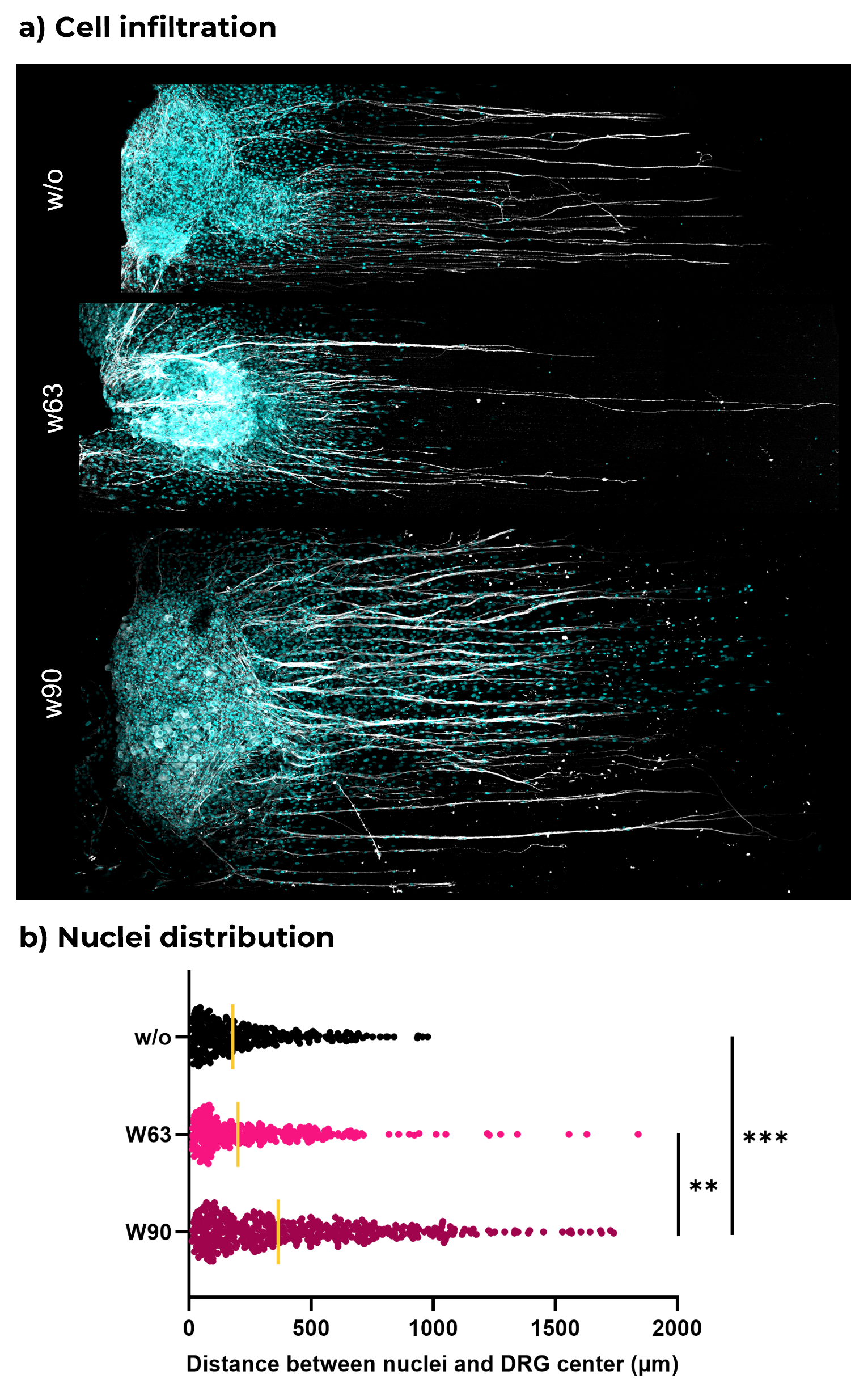


**Figure S4.** The macrochannels promote the cell infiltration. A) Representative confocal images of nuclei (cyan) and neurite (gray); and b) Distribution of the distance between nuclei and DRG center in varying matrices (n=3, dataset size > 278).


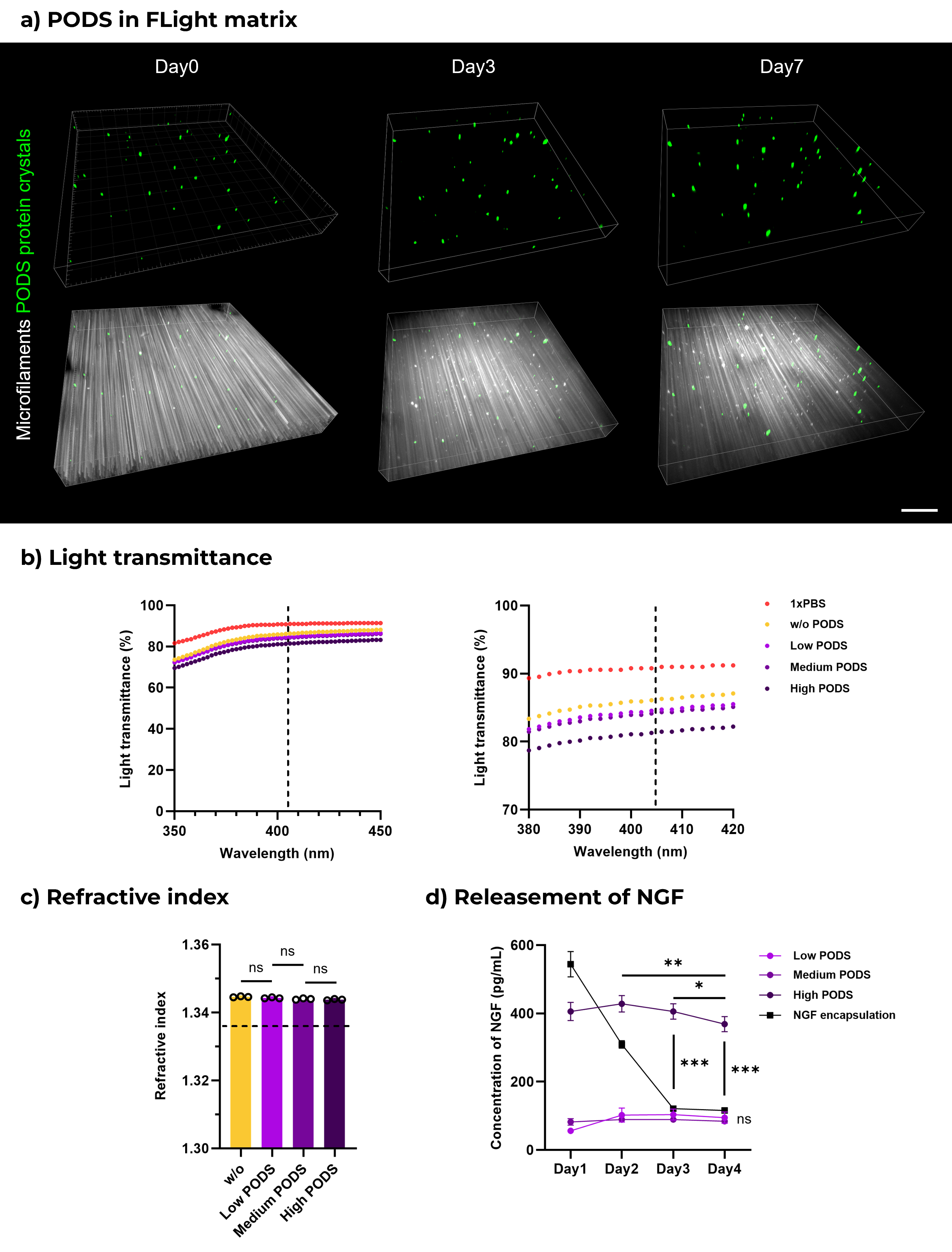


**Figure S5. Characterization of PODS-laden photoresin and releasement of protein cargo. a**) Representative 3D views indicated that GFP-labeled PODS were encapsulated in FLight matrix (high concentration) and maintained after 0, 3, and 7 days of culture. Scale bar: 100 µm. **b-c**) Characterization of photoresin mixed with different concentrations of NGF-PODS. The dashed line in the light transmittance panel indicates the wavelength of the laser utilized in the FLight system. The dashed line in Figure c represents the refractive index of 1x PBS. **d**) The assessment of NGF release from PODS into the culture medium on days 1, 2, 3, and 4. As a control, an equivalent total amount of NGF (matching the highest encapsulation concentration in NGF-PODS) was mixed with photoresin to print FLight hydrogel samples.


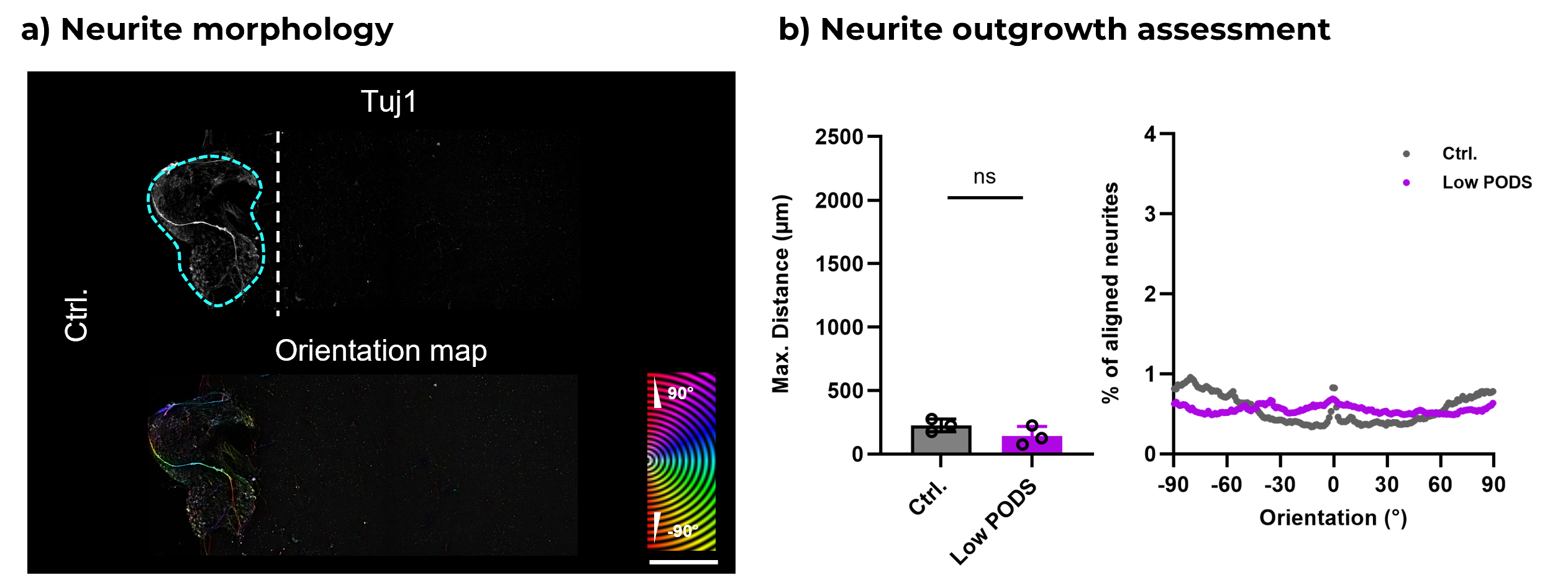


**Figure S6. Neurite outgrowth in 3D FLight matrices with and without NGF-PODS. a)** Representative confocal image of neurite outgrowth in FLight hydrogels without encapsulation of NGF-PODS (Ctrl.), with no additional NGF in the culture medium. The orientation map was generated from Tubulin Beta III (Tuj1) signal. Scale bar: 500 µm. **b**) The maximum outgrowth distance and alignment measurement of neurites in Ctrl. group vs. hydrogel matrices containing a low concentration of NGF-PODS. In both conditions, no additional NGF was added to the culture medium.


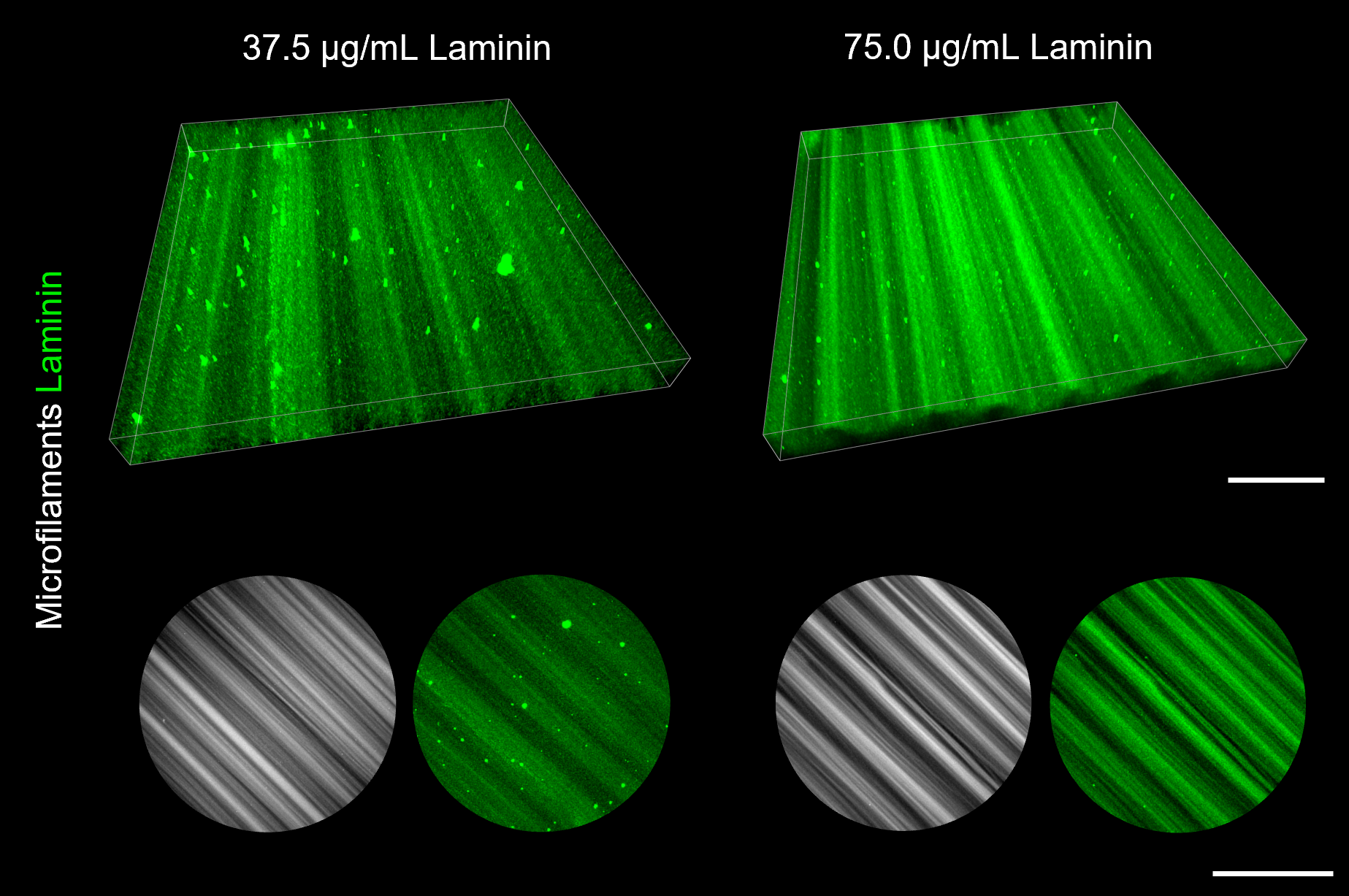


**Figure S7.** Confocal images of laminin (LN) and fluorescently labeled microfilaments in FLight matrices after 4 days of culture. Scale bar: 50 µm.


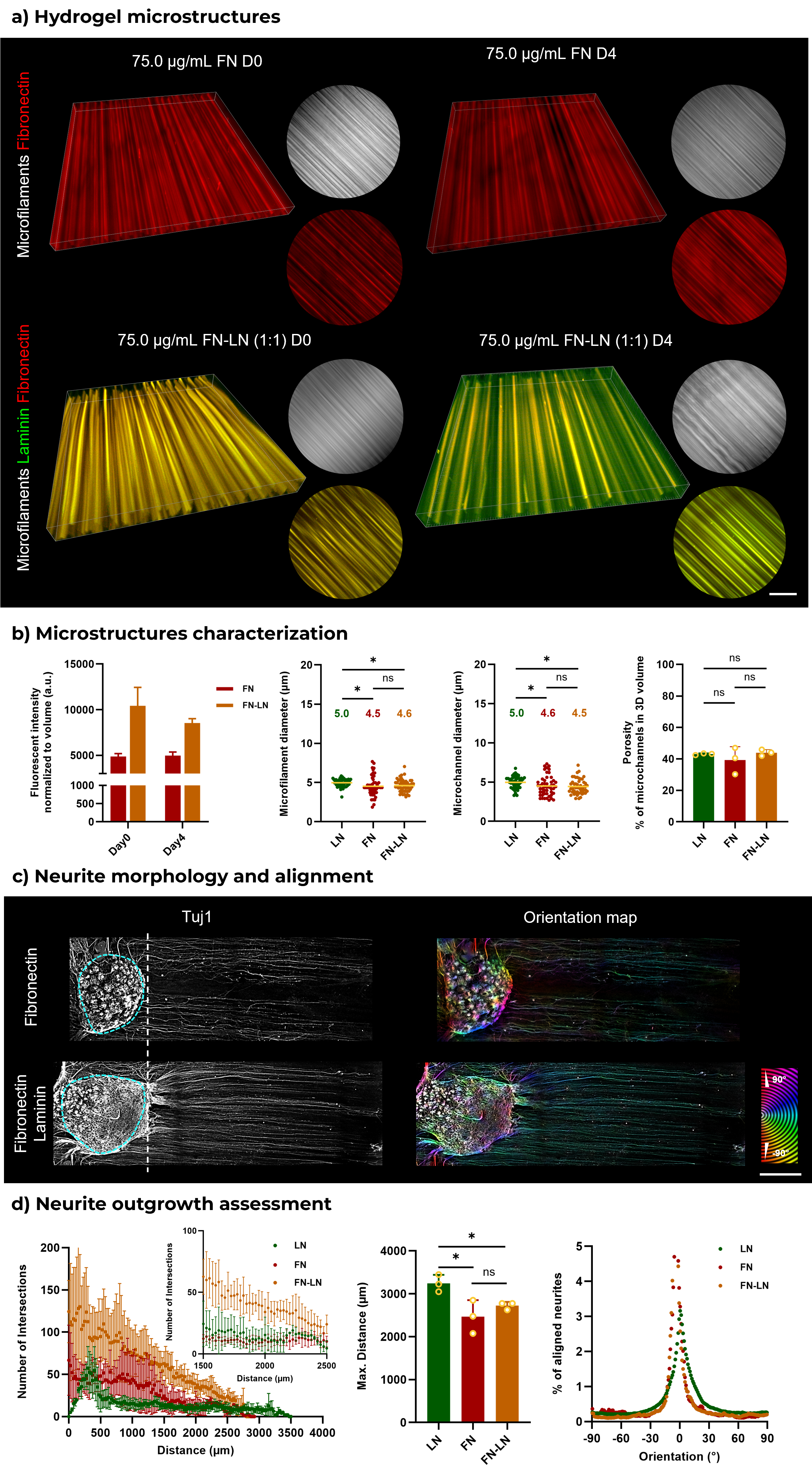


**Figure S8. Neurite outgrowth supported by bioactive molecules in 3D FLight hydrogel.** **a**) Confocal microscopy images showing fibronectin (FN) and a fibronectin-laminin mixture (FN-LN) in 3D FLight matrices at a total molecule concentration of 75 µg/mL at different time points. Scale bar: 100 µm. **b**) Normalized fluorescence intensity of FN and FN-LN from confocal images (left), along with quantification of microstructure dimensions in 3D FLight hydrogel (n=3, sample size: 50). Numbers depict the mean value of microstructure dimensions. **c**) Representative confocal images of neurite outgrowth in FLight hydrogels containing FN and FN-LN. Blue dashed lines highlight the dorsal root ganglion (DRG) body. An orientation map was generated using Tubulin Beta III (Tuj1) staining. Scale bar: 500 µm. **d**) Sholl analysis and alignment measurement of neurites in LN-laden and FN-laden samples, as well as FLight matrices combined with FN and LN (FN-LN) (n=3).


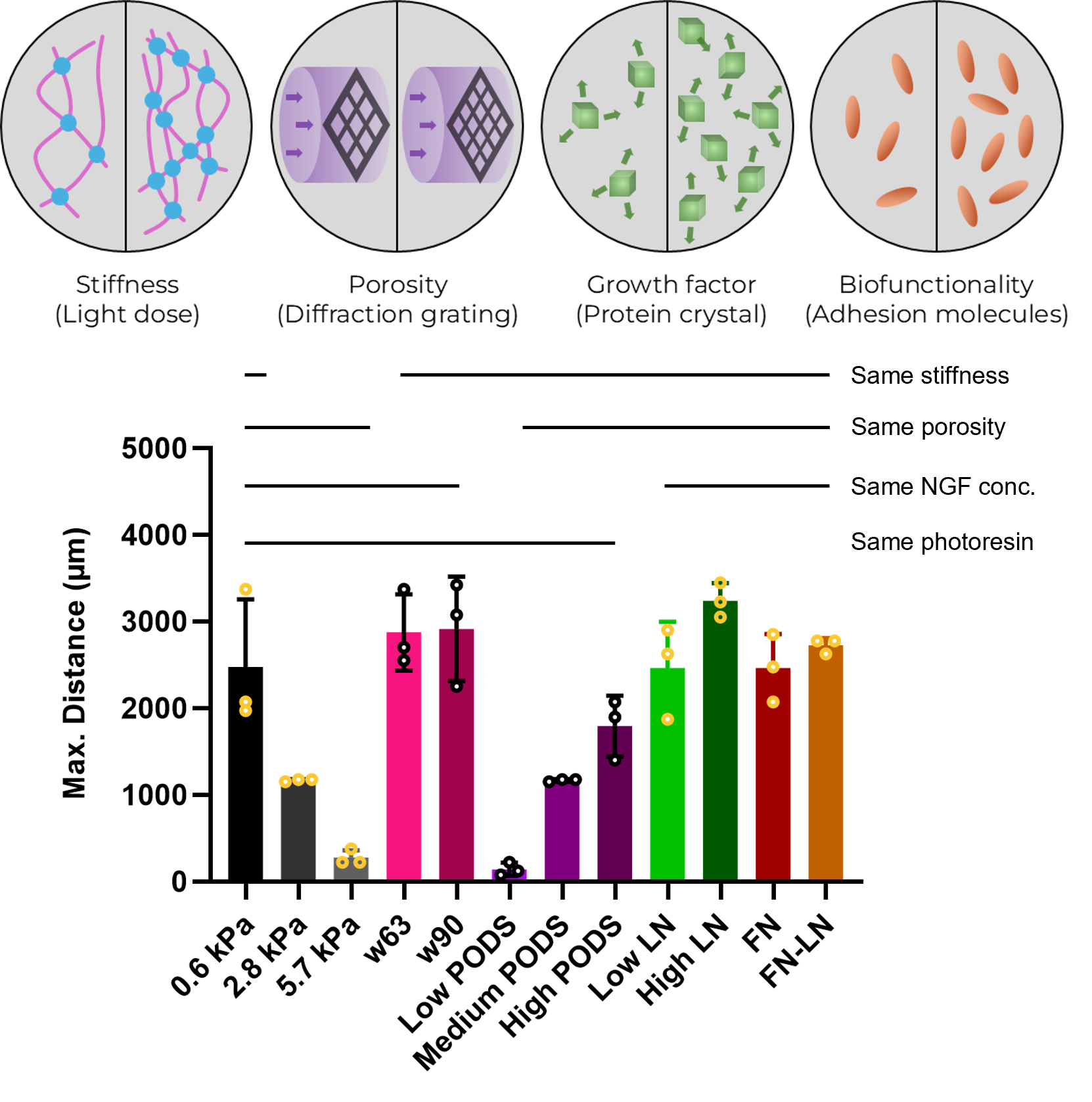


**Figure S9. Summary of maximum neurite outgrowth distances in tunable 3D FLight hydrogel matrices with varied properties.**

**References:**

[1] M. Singh, A simple experiment to study the array theorem using Fraunhofer diffraction of a two-dimensional grating, Phys Educ 58 (2023) 45007. https://doi.org/10.1088/1361-6552/acd5a3.
